# Effect of Heat treatment parameters on the tribological performance of LM26/graphite/fly ash hybrid aluminium matrix composites using Taguchi-TOPSIS optimization

**DOI:** 10.1371/journal.pone.0360156

**Published:** 2026-10-09

**Authors:** Prakash Chellamuthu, Palanisamy Angappan, Palanisamy Duraisamy, Abhishek Agarwal, Karma Dorji

**Affiliations:** 1 Department of Mechanical Engineering, Roever Engineering College, Perambalur, TamilNadu, India; 2 Department of Mechanical Engineering, Surya Engineering College, Erode, TamilNadu, India; 3 Department of Mechanical Engineering, Adhi College of Engineering & Technology, Kancheepuram, TamilNadu, India; 4 Department of Energy Technology, Tallinn University of Technology, Tallinn, Estonia; 5 Department of Mechanical Engineering, College of Science and Technology, Royal University of Bhutan, Phuentsholing, Bhutan; Institute of Infrastructure Technology Research and Management, INDIA

## Abstract

Hybrid aluminium matrix composites (HAMMCs) are of interest for lightweight components requiring improved resistance to wear and friction. The present study investigates the effects of solution treatment temperature, holding time and cooling method on the tribological behaviour of stir-cast LM26/graphite/fly ash hybrid aluminium matrix composites. A Taguchi L27 orthogonal array was used to evaluate solution treatment temperatures of 450, 500 and 550 °C, holding times of 2, 4 and 6 h, and furnace cooling, air cooling and water quenching. Hardness, wear rate and coefficient of friction (COF) were considered as response characteristics. TOPSIS was used for multi-response evaluation, and analysis of variance (ANOVA) was used to assess the contribution of the heat treatment parameters. Scanning electron microscopy (SEM) was used to examine reinforcement distribution in the matrix. The measured hardness ranged from 42.3 to 51.2 BHN, while the wear rate and COF ranged from 0.000667 to 0.000750 mm^3^/min and 0.4002 to 0.4501, respectively. The experimental results showed that hardness increased from 42.3 to 51.2 BHN, while wear rate and coefficient of friction decreased from 0.000750 to 0.000667 mm^3^/min and from 0.4501 to 0.4002, respectively, across the investigated conditions. Experiment 18, corresponding to 500 °C, 6 h and water quenching, gave the highest TOPSIS relative closeness value (0.8933). Factor-level analysis identified 550 °C, 6 h and water quenching (A3B3C3) as the optimum combination within the investigated design space. Temperature had the greatest influence on wear rate, coefficient of friction and the TOPSIS grade, whereas cooling method had the greatest influence on hardness. These results show that the selected heat treatment conditions substantially affected the combined hardness–wear–friction response of the hybrid composite under the investigated dry-sliding conditions.

## Introduction

Aluminium alloys are widely employed in aerospace, automotive and engineering applications owing to their high strength-to-weight ratio, excellent corrosion resistance and favourable tribological properties [1]. Heat treatment is one of the most effective post-processing techniques for improving the mechanical and wear performance of aluminium alloys and their composites by modifying the microstructure and strengthening the matrix [2]. Previous studies have demonstrated that heat treatment parameters, including solution treatment temperature, holding time and cooling method, significantly influence hardness, wear resistance and fatigue behaviour, although the optimum conditions depend on the alloy composition, reinforcement system and service requirements [3]. The effect of aging treatment on Selective Laser Melting (SLM) aluminium composites has also been examined, revealing enhancements in hardness and tensile strength due to grain refinement and residual stress relief [4]. Furthermore, the influence of T6 heat treatment on LM25 aluminium composites reinforced with TiB_2_, WC, and ZrO_2_ demonstrated that WC-reinforced composites exhibit superior mechanical properties and reduced wear rates after aging treatment [5]. Fly ash-reinforced aluminium composites have attracted considerable attention because of their low cost and improved mechanical characteristics, and heat treatment has been reported to further enhance their physical and mechanical properties through microstructural modification [6]. In addition, investigations on SLM-fabricated 7075 aluminium alloy subjected to different heat treatment conditions revealed notable improvements in microstructure, hardness, and corrosion resistance, with hardness values increasing to an average of HV123 compared with the as-manufactured alloy [7]. Among cast aluminium alloys, LM26 is particularly attractive because of its excellent castability, corrosion resistance and favourable strength-to-weight ratio, making it suitable for lightweight components subjected to sliding wear. Al-Si aluminium alloys are used in components where low mass, castability and adequate wear performance are required, including engine blocks, pistons and cylinder liners. Aluminium-based alloys and composites are used across automotive, aerospace, electronic and other engineering applications because their low density and tailorable mechanical, thermal and tribological properties allow them to meet different performance requirements [8–11]. Aluminium 6061 provides another example in which particulate reinforcement can be used to modify the performance of the base alloy. Although Al6061 offers low density and good corrosion resistance, its impact toughness can limit its performance under sudden dynamic loading. Recent work has therefore investigated the incorporation of particulate reinforcement to modify the microstructure, hardness and impact response of Al6061 [12]. Their application is associated with reductions in component mass and, consequently, potential energy savings, while the silicon-rich structure can provide useful resistance to sliding wear. Aluminium-based composites extend these advantages by incorporating hard or lubricating reinforcement phases to tailor hardness, friction and wear behaviour for specific service conditions [8,9]. However, its tribological performance can still be further improved through appropriate reinforcement and heat treatment [13].

The effect of heat treatment on the corrosion behaviour of Selective Laser Melting (SLM)-processed Al-Mg-Sc-Zr alloy was investigated through electrochemical measurements, and the SLM-fabricated specimens exhibited enhanced corrosion resistance after heat treatment [14]. The influence of T6 heat treatment on metakaolin-reinforced AA6082 aluminium composites demonstrated that the combined effect of reinforcement addition and heat treatment significantly improved both mechanical properties and wear resistance [15]. In another study, T6 treatment and the addition of 0.6 wt.% (Pr + Ce) in AlSi5Cu1Mg alloy refined the α-Al and eutectic Si phases and promoted a more uniform distribution of the Al_2_Cu phase, resulting in improved microhardness and corrosion performance [16].

Similarly, investigations on coarse and fine zircon particle-reinforced LM3 aluminium composites revealed that T6 heat treatment increased hardness by 13% and 15%, respectively, compared with non-heat-treated composites, while also considerably enhancing wear resistance [17]. Studies on AA7075-TiC aluminium composites further confirmed that T6 treatment effectively improves mechanical strength and tribological behaviour. Optimal solution treatment at 495°C for 8 h followed by aging at 205°C for 6 h produced higher strength and hardness, although a reduction in ductility was observed relative to the solution-treated and as-cast conditions [18].

The optimization of the heat treatment cycle for Al6082 alloy identified 495°C for 8 h as the optimum solution treatment temperature and 205°C for 6 h as the optimum artificial aging condition. Under these parameters, significant improvements in tensile strength and hardness were achieved, accompanied by a slight increase in impact energy and a moderate reduction in elongation, demonstrating the critical role of tailored heat treatment in achieving the desired balance of mechanical properties [19]. Similarly, T6 heat treatment of WC-reinforced LM28 aluminium composites produced substantial enhancements in hardness and wear resistance compared with T4-treated and untreated composites. Wear testing under different loads and sliding distances revealed reduced surface damage and lower frictional effects in the T6 condition, indicating the suitability of these composites for high-wear applications [20]. Al6Mg-based self-lubricating composites reinforced with graphite and fly ash were successfully fabricated through stir casting. Increasing the graphite content improved mechanical strength while simultaneously reducing the coefficient of friction and wear rate, and SEM analysis confirmed the dominant wear mechanisms associated with the presence of solid lubricants [21,22].

Hot-pressed powder metallurgy was employed to produce aluminium composites containing up to 18% graphite. Although densification decreased with increasing graphite content, the composite with 18% graphite exhibited enhanced thermal conductivity and reduced thermal expansion. Thermal cycling studies revealed crack formation at the matrix-graphite interface due to differences in coefficients of thermal expansion, which agreed well with theoretical predictions [23,24].

Aluminium matrix composites reinforced with 3–10% SiC microparticles were fabricated using both conventional stir casting and ultrasonic-assisted stir casting. The ultrasonic-assisted process achieved a more uniform distribution of SiC particles, thereby overcoming the limitations of conventional stir casting and resulting in improved mechanical properties, enhanced physical characteristics, and optimized density [25]. Tribological optimization of A356-10 wt.% SiC-Graphite hybrid aluminium composites was performed using the Taguchi method, Grey Relational Analysis (GRA), and TOPSIS. The optimum condition of 40 N load, 1 m/s sliding speed, and 3 wt.% graphite produced the minimum wear rate and coefficient of friction. ANOVA identified normal load as the most influential parameter, while SEM analysis revealed adhesive wear and delamination as the dominant wear mechanisms [26–28].

A matrix material selection framework for aluminium hybrid metal matrix composites was developed using Fuzzy AHP and TOPSIS, considering tensile strength, hardness, melting point, density, and cost as evaluation criteria. Both analytical and experimental results identified AA7075 as the most suitable matrix material, demonstrating the reliability of the proposed Fuzzy AHP-TOPSIS approach [29]. A hybrid AHP-TOPSIS multi-criteria decision-making framework was proposed to select the most suitable aluminium alloy series for MMC casting by considering mechanical, thermal, processing, and cost-related criteria. Sensitivity analysis confirmed the stability of the ranking results, demonstrating the reliability of the proposed model for aluminium matrix material selection [30,31].

Eco-friendly Al7075-T6 hybrid metal matrix composites reinforced with SiC, crumb rubber (CR), and MoS_2_ were fabricated through the stir casting process, and the process parameters were optimized using the Taguchi L18 design. TOPSIS validation confirmed the optimal process parameters and the improved mechanical performance of the developed green hybrid composite [32]. The machining performance of hybrid Al7075/B_4_C/Gr composites during Abrasive Aqua Jet Machining was optimized using RSM-CCD and ANOVA by evaluating water jet pressure, stand-off distance, and traverse speed. TOPSIS-based multi-objective optimization identified the optimum condition of 210 bar water jet pressure, B1 stand-off distance, and 30 mm/min traverse speed, resulting in an MRR of 4.8703 mm^3^/min, surface roughness of 3.57 μm, and taper angle of 0.189°, with a 49.6% improvement in taper angle compared with single-objective optimization [33]. The selection of a suitable matrix material for AHMMCs was performed using an integrated Fuzzy AHP-TOPSIS approach considering tensile strength, hardness, melting point, density, and cost. The analysis identified AA7075 as the most appropriate matrix material due to its superior overall performance [34,35]. The mechanical and wear behaviour of stir-cast Al-Mg-Si composites reinforced with steel, steel-graphite hybrid, and SiC particles was investigated. Steel reinforcement improved hardness, tensile strength, specific strength, and fracture toughness, while the steel-graphite hybrid reinforcement provided the highest wear resistance, indicating a balanced combination of mechanical and tribological performance [35]. Al-xGnP nanocomposites fabricated by powder metallurgy showed improved wear resistance up to 3 wt.% xGnP, with maximum hardness at 1 wt.% xGnP. However, tensile strength decreased with increasing xGnP content due to the formation of brittle Al_4_C_3_ phases at the interface [36,37]. AA6082 composites reinforced with 0–12 wt.% graphite particles exhibited reductions in hardness, tensile strength, and elongation with increasing graphite content. Although density decreased, porosity increased as the graphite reinforcement level increased [38]. Al6082-graphite composites containing 2–6 wt.% graphite were fabricated by a two-stage stir casting process. Uniform graphite distribution improved tensile strength and significantly enhanced wear resistance, although a slight decrease in hardness was observed [39].

Heat-treated AA7075-Si_3_N_4_ composites produced by electromagnetic stir casting showed maximum tensile and flexural strength at 8 wt.% Si_3_N_4_, while 12 wt.% Si_3_N_4_ provided the highest wear resistance and reduced the coefficient of friction. Regression models accurately predicted wear and friction behaviour [40]. AA7075-SiC-Al_2_O_3_ hybrid composites exhibited uniform reinforcement distribution, with optimum hardness at 10 wt.% reinforcement. T6 heat treatment further increased hardness by 24% compared with the as-cast condition [41]. Heat-treated LM25/AlB_2_ composites achieved a maximum hardness of 82 HRB after aging at 250°C for 6 h. Taguchi and ANOVA analyses identified sliding velocity as the most significant factor affecting wear rate [42].

Recent studies have explored the development of Al7075-based composites reinforced with high-entropy alloy particles (HEAp) using ultrasonic-assisted stir casting. The incorporation of an optimum reinforcement content of 6 wt.% HEAp, combined with heat treatment, resulted in significant improvements in tensile strength and hardness, whereas excessive reinforcement adversely affected the mechanical properties [43]. In another investigation, stir-cast hybrid aluminium composites subjected to post-fabrication heat treatment exhibited notable changes in wear behaviour, indicating that heat treatment plays a crucial role in enhancing the tribological performance of hybrid metal matrix composites [44]. Different reinforcement phases provide different combinations of properties. SiC and Al_2_O_3_ are widely used where increased hardness, stiffness and wear resistance are required, while B_4_C is attractive for applications requiring high hardness and wear resistance. TiC is also used as a hard particulate reinforcement in aluminium matrix composites. Graphite differs from these ceramic reinforcements because it can act as a solid lubricant and reduce friction, although excessive graphite addition may adversely affect hardness. Fly ash provides a low-cost particulate reinforcement whose ceramic constituents can contribute to hardness and wear resistance, while its composition and particle morphology may vary with source [45,46]. The principal advantage of aluminium matrix composites is the ability to combine low density with tailored mechanical and tribological properties; their limitations include particle agglomeration, porosity, processing complexity and possible loss of ductility at high reinforcement contents. Overall, previous studies indicate that reinforcement type and heat treatment can produce different balances between hardness, strength, friction and wear resistance. Hard ceramic reinforcements such as SiC, TiC and Al_2_O_3_ generally favour hardness and wear resistance, whereas graphite can reduce friction because of its lubricating action. Hybrid reinforcement can therefore provide a balance between mechanical and tribological properties, but the resulting response depends on the alloy, reinforcement content and test conditions [26]. These differences indicate that the optimum material condition cannot be transferred directly between aluminium composite systems and should instead be established for the specific alloy, reinforcement combination and processing conditions investigated.

Although aluminium matrix composites have been extensively studied, limited work has examined the combined influence of solution-treatment temperature, holding time, and cooling method on the hardness, wear rate, and coefficient of friction of an LM26/graphite/fly-ash hybrid composite using one common experimental design [40–44]. The present study addresses this specific gap by evaluating a fixed LM26/10 wt.% graphite/5 wt.% fly-ash composition under dry-sliding conditions. Taguchi factor-level analysis and TOPSIS were used to compare hardness, wear rate, and coefficient of friction simultaneously, while ANOVA was used to estimate the relative effects of the three heat-treatment factors within the investigated parameter range. The study identifies a suitable condition for the tested composite and test configuration; it does not propose a universal optimum for aluminium matrix composites.

The objective of this study was to determine how solution-treatment temperature, holding time, and cooling method affect the measured hardness, wear rate, and coefficient of friction of the developed hybrid composite. A secondary objective was to compare the multi-response TOPSIS result with the individual response trends and to quantify the relative influence of the selected factors using a main-effects ANOVA model.

## Materials and methods

### Matrix material: LM26 aluminium alloy

LM26 aluminium alloy was selected as the matrix material owing to its good castability, corrosion resistance, low density and favourable strength-to-weight ratio. These characteristics make LM26 suitable for automotive and engineering components subjected to moderate mechanical and tribological loading [47]. The chemical composition and properties of the alloy were obtained from the material supplier and used as the basis for composite fabrication. The LM26 alloy contained 6.3 wt.% Si, 0.25 wt.% Mg, 0.45 wt.% Fe, 0.10 wt.% Cu, 0.25 wt.% Mn, 0.10 wt.% Ni, 0.05 wt.% Zn and 0.02 wt.% Ti, with Al as the balance. The hybrid composite contained 10 wt.% graphite and 5 wt.% fly ash. The graphite particles had an approximate size of 300 µm (50 mesh), while the fly ash particles were in the range of 50–80 µm. The graphite particles exhibited an irregular, flake-like morphology, while the fly ash particles appeared predominantly irregular and approximately equiaxed in the SEM micrograph. These observations were used to assess the morphology and distribution of the reinforcement particles within the aluminium matrix. Graphite was selected for its solid-lubricating behaviour, whereas fly ash was selected as a particulate reinforcement because its ceramic constituents can contribute to hardness and wear resistance. LM26 was chosen specifically because the study employs a casting route and examines post-casting heat treatment of a cast aluminium composite. The selection was therefore based on its castability and relevance to automotive and general engineering applications, rather than on a claim that LM26 is superior to other aluminium alloys.

### Fabrication of composites

LM26 aluminium alloy was melted in a graphite-coated crucible using an electric resistance furnace. Mechanical stirring was carried out using a ceramic-coated impeller operating at 400 rpm for 15 min to obtain a homogeneous melt. The required quantities of graphite and fly ash were weighed separately and preheated to 450 °C before addition to the molten alloy. Graphite was selected as a solid lubricant to reduce friction during sliding, whereas fly ash was selected as a hard ceramic reinforcement to improve hardness and wear resistance [48,49]. The reinforcements were preheated to remove moisture and improve their wettability with the aluminium melt. Approximately 1 wt.% magnesium was added to improve wettability between the molten aluminium alloy and the reinforcement particles, thereby reducing particle agglomeration and promoting more uniform distribution within the matrix. The preheated reinforcements were gradually introduced into the molten alloy under continuous stirring. Hexachloroethane degassing tablets were added prior to casting to minimise dissolved gases and reduce porosity in the cast composite. The molten composite slurry was then poured into a preheated metallic mould and allowed to solidify under ambient conditions before machining the specimens for subsequent heat treatment and tribological testing.

The stir-casting route was selected because it permits direct incorporation of particulate reinforcements into the molten aluminium matrix using relatively simple processing equipment and is suitable for producing particulate-reinforced aluminium composites. Compared with powder-based processing routes, it avoids separate powder compaction and sintering stages. Controlled stirring, reinforcement preheating and magnesium addition were used to address the main processing challenges of particle wetting and dispersion. A quantitative economic comparison of alternative processing routes was not performed in the present study; therefore, no specific cost advantage is claimed. The available experimental record does not contain sufficiently documented values for the melt temperature during reinforcement addition, reinforcement addition rate, impeller dimensions or geometry, mould temperature, or the quantity and duration of degassing. These parameters are therefore not assigned retrospectively. The fabrication description is limited to the parameters recorded in the experimental documentation.

Fig 1 illustrates the experimental setup used for stir casting, heat treatment and tribological testing of the fabricated composites. Fig 2 presents a representative SEM micrograph showing the distribution of graphite and fly ash reinforcements within the aluminium matrix. The graphite particles exhibited an irregular, flake-like morphology, while the fly ash particles appeared predominantly irregular and approximately equiaxed in the SEM micrograph. These observations were used to assess the morphology and distribution of the reinforcement particles before evaluating the heat-treatment effects. A separate microstructural image of the unreinforced LM26 alloy was not available in the experimental dataset used for the present study. Therefore, the manuscript reports the SEM evidence available for the fabricated hybrid composite rather than introducing an unverified base-alloy microstructure.

**Fig 1:**
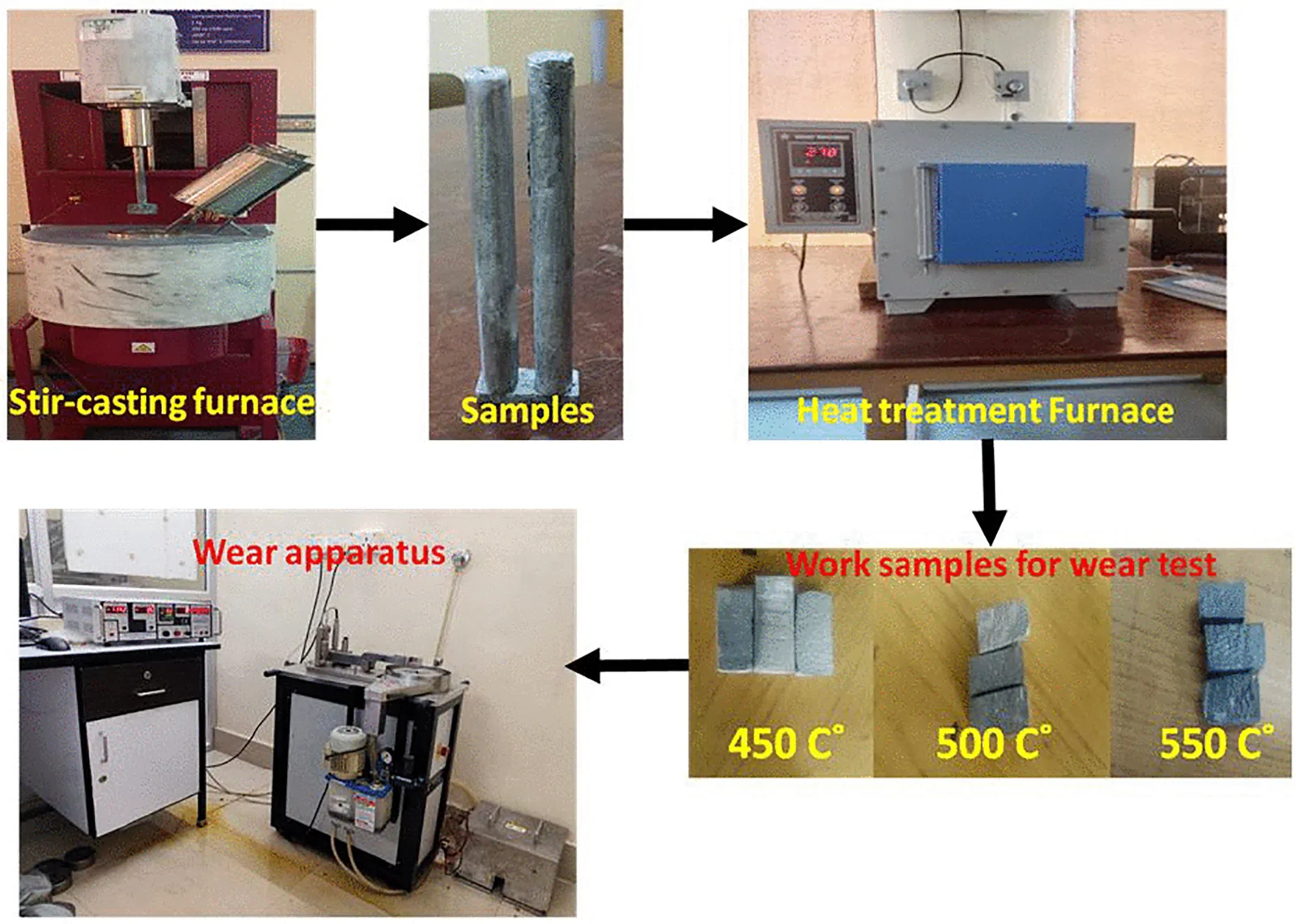
Experimental setup for stir casting, heat treatment and dry sliding wear testing.

**Fig 2:**
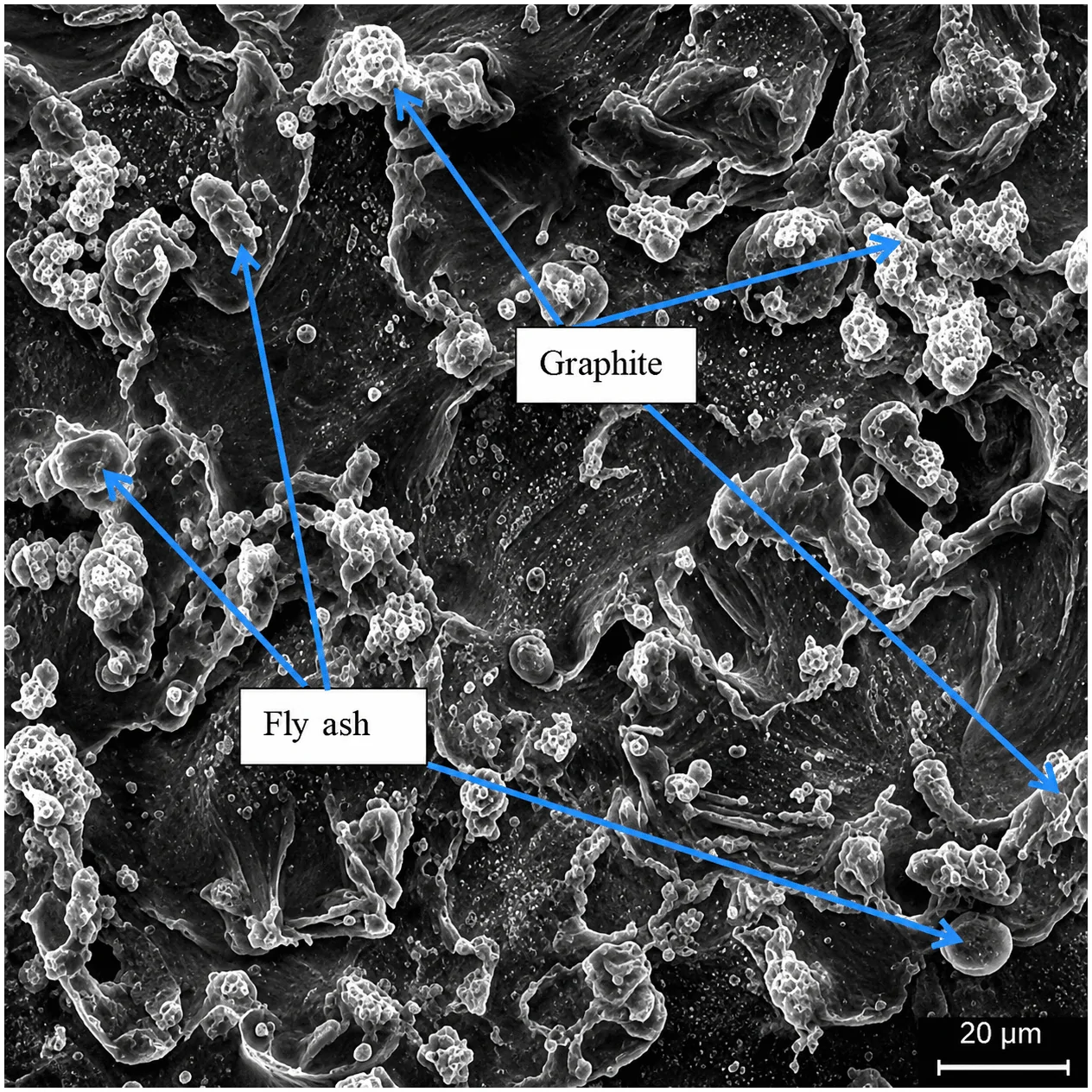
SEM micrograph showing the distribution of graphite and fly ash particles within the LM26 aluminium matrix.

The experimental programme comprised 27 treatment conditions generated using a Taguchi L27 orthogonal array. Three factors were varied at three levels: solution-treatment temperature (450, 500 and 550 °C), holding time (2, 4 and 6 h), and cooling method (furnace cooling, air cooling and water quenching). Each experimental condition represented a unique factor-level combination, and the resulting specimens were evaluated for hardness, wear rate and coefficient of friction. One experimental result was reported for each of the 27 factor combinations in the available dataset. Thus, the experimental programme comprised 27 reported observations corresponding to the 27 unique heat-treatment conditions for the selected LM26/10 wt.% graphite/5 wt.% fly-ash composite. No separate material compositions were evaluated in this study. Independent replicate specimens were not reported for the individual factor combinations, and therefore within-condition variability and repeatability cannot be estimated from the available experimental data. The measured responses were subsequently used for TOPSIS analysis and ANOVA. The complete experimental dataset is provided in Supporting Information (S1 Data). The Taguchi L27 orthogonal array was selected to systematically evaluate the three heat-treatment factors at three levels and to permit their main effects to be evaluated systematically.

The specimens were solution treated at 450, 500 and 550 °C for 2, 4 and 6 h according to the combinations specified in Table 1. The selected temperature range of 450–550 °C and holding times of 2–6 h were chosen based on solution-treatment conditions reported for cast aluminium alloys and aluminium matrix composites [5,6,14,38]. The three temperature and holding-time levels were selected to provide a systematic assessment of increasing treatment severity within the investigated experimental range rather than to establish a phase-transformation boundary for LM26. The three holding times represent short, intermediate and extended treatment durations within the selected range. After treatment, the specimens were cooled using furnace cooling, air cooling or water quenching. Artificial ageing was not included because the objective was to evaluate the effects of solution-treatment temperature, holding time and cooling method without introducing a subsequent ageing step. Accordingly, the results should not be interpreted as a direct comparison of conventional T4 or T6 ageing schedules.

**Table 1:** Heat treatment parameters and their levels.

Parameters	Level 1	Level 2	Level 3
Temperature (°C)	450	500	550
Time (h)	2	4	6
Cooling Method	(1) Furnace cooling	(2) Air cooling	(3) Water quenching

### Tribological testing

Dry sliding wear tests were performed using a pin-on-disc tribometer under dry conditions. The available experimental record does not identify a specific ASTM or ISO standard for the tribological measurements. The tests were nevertheless performed using the same pin-on-disc configuration and operating procedure for all experimental conditions, allowing comparative assessment of the effect of the selected heat-treatment factors. Previous studies have similarly emphasized controlled experimental conditions together with systematic wear and material characterization when evaluating the influence of processing conditions on tribological performance [48,49]. The tribological configuration was selected to provide a controlled dry-sliding comparison of the heat-treated composite conditions rather than to reproduce one specific service tribosystem. Consequently, the measured responses are intended to indicate relative changes in sliding wear and friction under a common laboratory configuration. Direct transfer of the measured values to a particular engineering contact should therefore be made with caution because service conditions such as lubrication, contact pressure, sliding velocity, counter-body material and surface finish may differ substantially.

The same test configuration and operating conditions were maintained for all 27 experimental conditions. The coefficient of friction (COF) was recorded during sliding, and the wear rate was determined from the measured material loss using the adopted calculation procedure. No lubricant was used during the tests. The same specimen configuration and test procedure were applied to all experimental conditions to provide a consistent basis for comparison. The available experimental record used for the present analysis does not contain sufficiently documented values for the counter-body geometry and material, applied load, sliding speed, sliding distance, wear-track dimensions, or specimen dimensions. These parameters are therefore not assigned retrospectively in the present manuscript. The reported wear rate and coefficient of friction should consequently be interpreted as comparative responses obtained under the same dry-sliding test configuration rather than as a complete tribological characterisation for a specific service system. Surface roughness was not measured or included as a response variable in the experimental dataset available for this study. Accordingly, roughness values for the 27 tested conditions cannot be reported retrospectively. This limitation is relevant because surface roughness can influence the initial sliding contact and wear response. Future experiments should therefore include controlled surface-finish measurements before tribological testing. The tribological test conditions were kept constant throughout the experimental programme so that the influence of heat-treatment parameters could be evaluated without introducing additional tribological variables. The overall experimental methodology, including composite fabrication, heat treatment, tribological testing, TOPSIS-based multi-response optimization and ANOVA, is illustrated in Fig 3.

**Fig 3:**
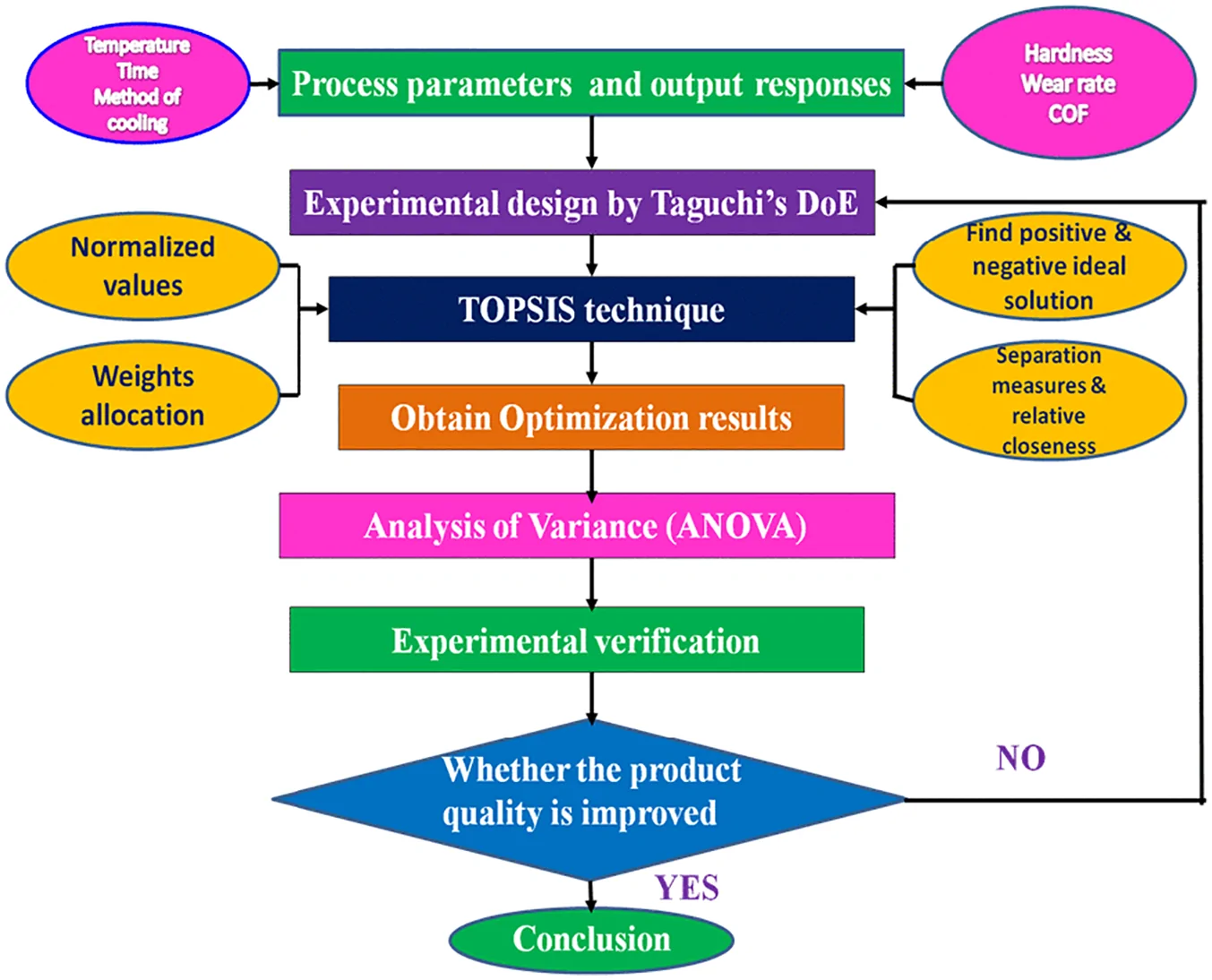
Flow chart of the experimental methodology, including TOPSIS-based optimization and ANOVA.

## Results and discussion

### Technique for Order Preference by Similarity to Ideal Solution (TOPSIS)

The Technique for Order Preference by Similarity to Ideal Solution (TOPSIS) is one of the most widely adopted multi-criteria decision-making (MCDM) methods for simultaneously optimizing multiple performance characteristics. The method identifies the optimum alternative based on the shortest distance from the positive ideal solution and the farthest distance from the negative ideal solution [29,30]. In the present study, TOPSIS was employed because it simultaneously considers beneficial (hardness) and non-beneficial (wear rate and coefficient of friction) responses, enabling the identification of a single optimum heat treatment condition for the twenty-seven experimental trials generated using the Taguchi L27 orthogonal array without assigning subjective preference to any individual response. The computational procedure adopted in this work is summarized in the following steps.

***Stage: 1***

The data have been normalized using the following relation:


$$\begin{array}{c} R_{ij=\frac{X_{ij}}{\sum X_{ij}^{\text{2}}}}\text{ for }i\;=\;\text{1}\ldots \text{ m};\;j\;=\;\text{1}\ldots \text{ n } \end{array}$$ (1)

***Stage: 2***

Weights were allocated for each criterion considered in optimization. Equal weights were assigned to the three response characteristics. A weighted normalized matrix was built by multiplying each column of the normalized matrix with the respective weights.


$$\begin{array}{c} V_{ij}=W_{j}R_{ij} \end{array}$$ (2)

Let *W*_j_ be the weights for each input parameter where *j* = 1…n. (equal to 0.333)

Equal weights of 1/3 were assigned to hardness, wear rate and coefficient of friction because no prior weighting scheme or application-specific preference was established among the three response characteristics. Hardness was treated as a beneficial response, whereas wear rate and coefficient of friction were treated as non-beneficial responses. A sensitivity analysis was performed using alternative weighting schemes in which one response was assigned a weight of 0.40 or 0.50 while the remaining weights were distributed equally among the other responses. The resulting rankings were compared with the equal-weight case to assess the stability of the optimum. The six alternative weighting schemes considered were (0.40, 0.30, 0.30), (0.30, 0.40, 0.30), (0.30, 0.30, 0.40), (0.50, 0.25, 0.25), (0.25, 0.50, 0.25), and (0.25, 0.25, 0.50) for hardness, wear rate, and coefficient of friction, respectively. Experiment 18 remained the highest-ranked condition under all six weighting schemes, indicating that the ranking of the best observed experimental condition was stable within the weighting range examined.

***Stage: 3***

Ideal and negative ideal solutions have been found using the relation given:

(i) Positive ideal solution.

*A*^*+*^ = {*V*_*1*_^*+*^*, …, V*_*n*_^*+*^}; where *V*_*j*_^*+*^={max *(V*_*ij*_*)* if *j* *J*; min *(V*_*ij*_*)* if *j* *J’*}

(ii) Negative Ideal solution.

*A*^*-*^ = {*V*_*1*_^*-*^*, …,V*_*n*_^*-*^}; where *V*_*j*_^*-*^ = {min *(V*_*ij*_*)* if *j* *J*; max *(V*_*ij*_*)* if *j* *J’*}

***Stage: 5***

The separation measure is found out from the positive and negative ideal solution using the relation given as follows:

The separation from the ideal alternative is:


$$\begin{array}{c} S_{i}^{+}{=[{\sum (V_{j}^{+}-V_{ij})}^{\text{2}}]}^{\text{1}/\text{2}}\;i\;=\;\text{1},\;\ldots ,\text{ m} \end{array}$$ (3)

Similarly, the separation from the negative alternative is:


$$\begin{array}{c} S_{i}^{-}{=[{\sum (V_{j}^{-}-V_{ij})}^{\text{2}}]}^{\text{1}/\text{2}}\;i\;=\;\text{1},\;\ldots ,\text{ m} \end{array}$$ (4)

***Stage: 6***

Relative closeness value of a particular alternative is found using the relation:


$$\begin{array}{c} P_{i}=\frac{S_{i}^{-}}{\left(S_{i}^{+}+S_{i}^{-}\right)}\;\text{0}<Pi\;<\;\text{1} \end{array}$$ (5)

The option with *P*_*i*_ closest to 1 is selected. The experimental conditions and measured responses for the 27 experimental trials are presented in Table 2. These values were used to identify the optimum heat treatment condition based on the overall tribological performance of the developed hybrid aluminium matrix composites.

**Table 2:** Experimental design of L_27_ OA with Heat treatment process parameters & corresponding measured outputs responses.

Ex. No.	Heat treatment parameters			Measured output responses		
	Temperature (°C)	Time (h)	Cooling Method (1. Furnace cooling 2. Air cooling 3. Water Quenching)	Hardness, (BHN)	Wear rate, (mm^3^/min)	COF
1	450	2	1	42.3	0.000750	0.4501
2	450	2	2	44.93	0.00074	0.444
3	450	2	3	46.96	0.000739	0.4434
4	450	4	1	43.8	0.000731	0.4387
5	450	4	2	45.1	0.000728	0.4366
6	450	4	3	47.23	0.000727	0.4362
7	450	6	1	44.49	0.000725	0.435
8	450	6	2	46.2	0.000721	0.4326
9	450	6	3	47.8	0.000715	0.429
10	500	2	1	44.72	0.000713	0.4278
11	500	2	2	46.8	0.000712	0.4272
12	500	2	3	47.8	0.000709	0.4254
13	500	4	1	45.3	0.000705	0.423
14	500	4	2	48.7	0.000703	0.4218
15	500	4	3	49.52	0.000701	0.4206
16	500	6	1	47.1	0.000683	0.4098
17	500	6	2	49.9	0.000679	0.4072
18	500	6	3	51.2	0.000681	0.4086
19	550	2	1	44.61	0.000699	0.4194
20	550	2	2	46.1	0.00069	0.414
21	550	2	3	47.5	0.000688	0.4128
22	550	4	1	45.2	0.000677	0.4070
23	550	4	2	47.72	0.000677	0.4062
24	550	4	3	48.3	0.000675	0.405
25	550	6	1	45.5	0.000673	0.4038
26	550	6	2	47.91	0.000671	0.4026
27	550	6	3	48.6	0.000667	0.4002

The normalized decision matrix weighted normalized matrix, separation measures, relative closeness values and corresponding TOPSIS rankings for the 27 experimental trials are presented in Table 3.

**Table 3:** TOPSIS relative closeness values and ranks for the 27 experimental conditions.

Ex. No.	Sᵢ⁺	Sᵢ^−^	Pᵢ	Rank
1	0.01625	0	0	27
2	0.01275	0.00384	0.2312	25
3	0.01096	0.00655	0.3742	21
4	0.01309	0.0032	0.1964	26
5	0.01147	0.0048	0.2948	23
6	0.00946	0.00739	0.4385	17
7	0.01186	0.00442	0.2713	24
8	0.00978	0.00653	0.4006	20
9	0.00775	0.0088	0.5316	15
10	0.01069	0.00582	0.3526	22
11	0.00837	0.00789	0.4851	16
12	0.00715	0.00922	0.5633	12
13	0.00946	0.00712	0.4295	18
14	0.00577	0.01068	0.649	9
15	0.00495	0.01176	0.7035	7
16	0.00599	0.01087	0.6447	10
17	0.00235	0.0139	0.8555	2
18	0.00181	0.01512	0.8933	1
19	0.00994	0.00731	0.4238	19
20	0.0076	0.00934	0.5514	13
21	0.00575	0.01072	0.6508	8
22	0.00835	0.01015	0.5488	14
23	0.00495	0.01201	0.7083	6
24	0.00411	0.01271	0.7556	4
25	0.00786	0.01087	0.5804	11
26	0.00454	0.01278	0.7377	5
27	0.00357	0.01377	0.7942	3

The sensitivity analysis showed that Experiment 18 remained the highest-ranked condition when the response weights were varied within the examined range, indicating that the identified ranking was not dependent on the equal-weight assumption. As shown in Table 3, Experiment 18 achieved the highest relative closeness value (0.8933) and was ranked first among the 27 experimental trials. This condition corresponds to a solution treatment temperature of 500 °C, a holding time of 6 h and water quenching. The result represents the best combined response of hardness, wear rate and coefficient of friction within the investigated experimental conditions.

### Heat treatment effects on the responses of Hardness, BHN

The measured hardness values of the developed hybrid aluminium matrix composites varied from 42.3 to 51.2 BHN, as presented in Table 2. The maximum hardness (51.2 BHN) was obtained at 500 °C, 6 h and water quenching (A2B3C3), whereas the minimum hardness (42.3 BHN) was recorded at 450 °C, 2 h and furnace cooling (A1B1C1). The measured increase in hardness indicates a clear effect of the heat treatment condition on the composite response. The available SEM observations confirm the presence and distribution of graphite and fly ash within the aluminium matrix, but they do not by themselves establish phase transformation, precipitation behaviour or changes in interfacial bonding after heat treatment. The higher hardness obtained after water quenching is therefore discussed as an experimental response rather than being assigned to a specific microstructural mechanism that was not directly characterized. The main effects plot shown in Fig 4 indicates that cooling method exerted the greatest influence on hardness, followed by solution treatment temperature and holding time.

**Fig 4:**
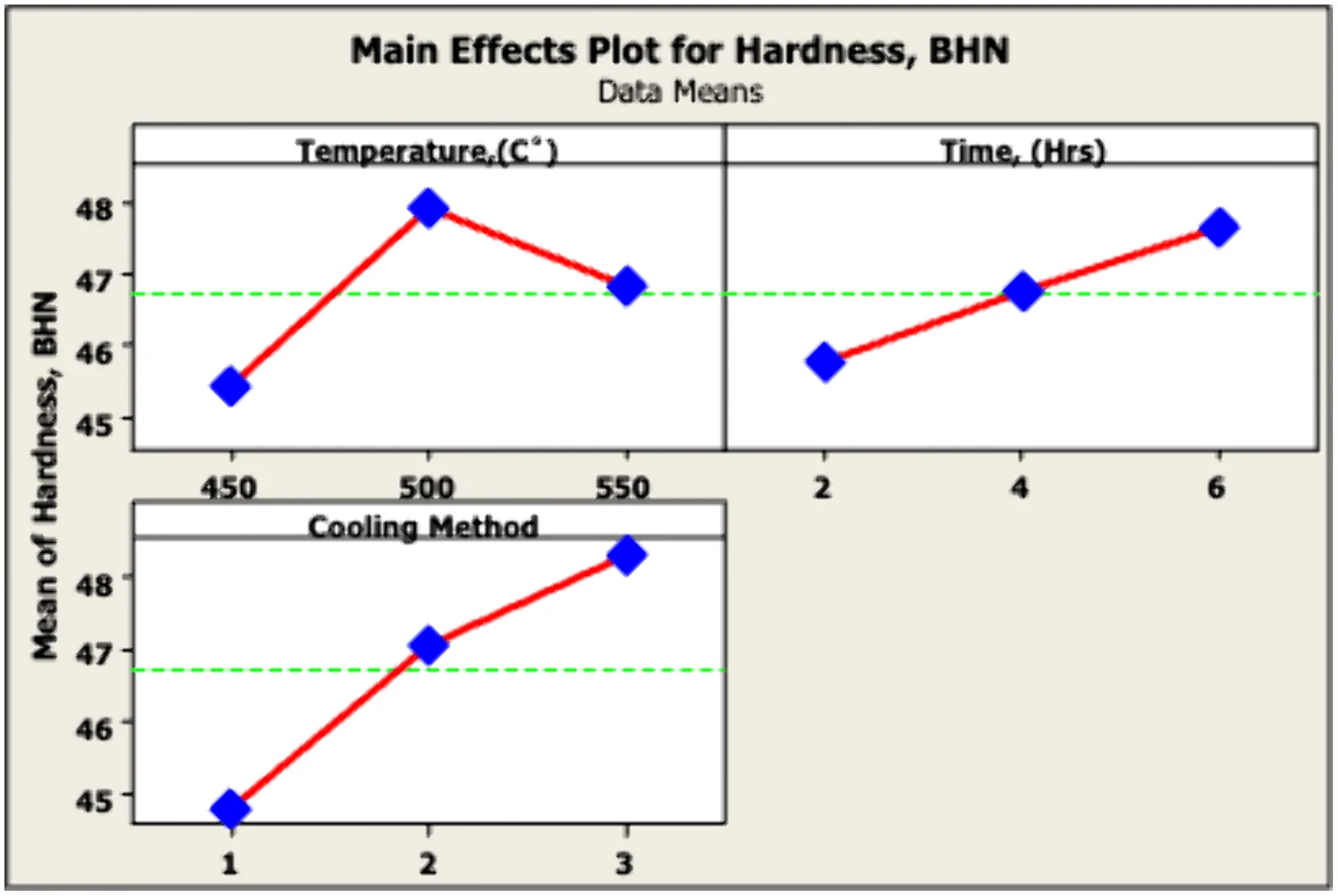
The main effect plots for the response of Hardness.

As illustrated in Fig 4, hardness increased with increasing holding time from 2 to 6 h, indicating that sufficient soaking promoted a more uniform microstructure before cooling. The highest average hardness was obtained under water quenching, whereas furnace cooling produced the lowest values because of its slower cooling rate. These observations are consistent with the hardness values reported in Table 2**.** The improvement in hardness agrees with previous investigations on heat-treated aluminium matrix composites, which attributed the enhancement to matrix strengthening and improved reinforcement-matrix interaction [17,20,41].

### Heat treatment effects on the responses of wear rate

The measured wear rate varied from 0.000667 to 0.000750 mm^3^/min, as presented in Table 2. The minimum wear rate (0.000667 mm^3^/min) was obtained at 550 °C, 6 h and water quenching (Experiment 27), whereas the maximum wear rate (0.000750 mm^3^/min) was observed at 450 °C, 2 h and furnace cooling (Experiment 1). The measured wear rate decreased with increasing solution-treatment temperature and holding time within the investigated range. The lowest average wear-rate response was obtained at the highest temperature and holding-time levels, while the effect of cooling method was comparatively smaller. Because post-wear SEM, EDS and subsurface characterization were not performed, the dominant wear mechanism, the extent of delamination and the formation of a mechanically mixed layer cannot be established directly from the present data. The observed reduction in wear rate can nevertheless be discussed in terms of the measured response and the known roles of the reinforcement phases: graphite can reduce interfacial shear through its solid-lubricating behaviour, while the harder fly-ash constituents can provide resistance to material removal. These effects are considered possible contributors rather than directly demonstrated wear mechanisms in the present study.

As illustrated in Fig 5**,** solution treatment temperature had the greatest influence on wear rate, followed by holding time and cooling method. Increasing the treatment temperature generally reduced the wear rate, indicating that appropriate heat treatment improved the resistance of the composite surface to material removal during sliding. This trend is consistent with the experimental observations presented in Table 2**.** A similar reduction in wear rate after heat treatment has been reported for heat-treated aluminium matrix composites reinforced with ceramic particles [20,41], confirming the beneficial effect of improved surface hardness on wear resistance. The present trends are also broadly consistent with earlier investigations of aluminium-based hybrid composites, in which heat treatment and reinforcement modification produced simultaneous changes in hardness, friction and wear behaviour [23,43,50]. The measured hardness range of 42.3–51.2 BHN, wear-rate range of 0.000667–0.000750 mm^3^/min and COF range of 0.4002–0.4501 therefore follow the general direction reported for reinforced aluminium systems, although direct numerical comparison should be made cautiously because alloy composition, reinforcement content, heat-treatment schedules and tribological test conditions differ between studies. The present results consequently support the reported trend rather than establishing equivalence with the performance of other composite systems.

**Fig 5:**
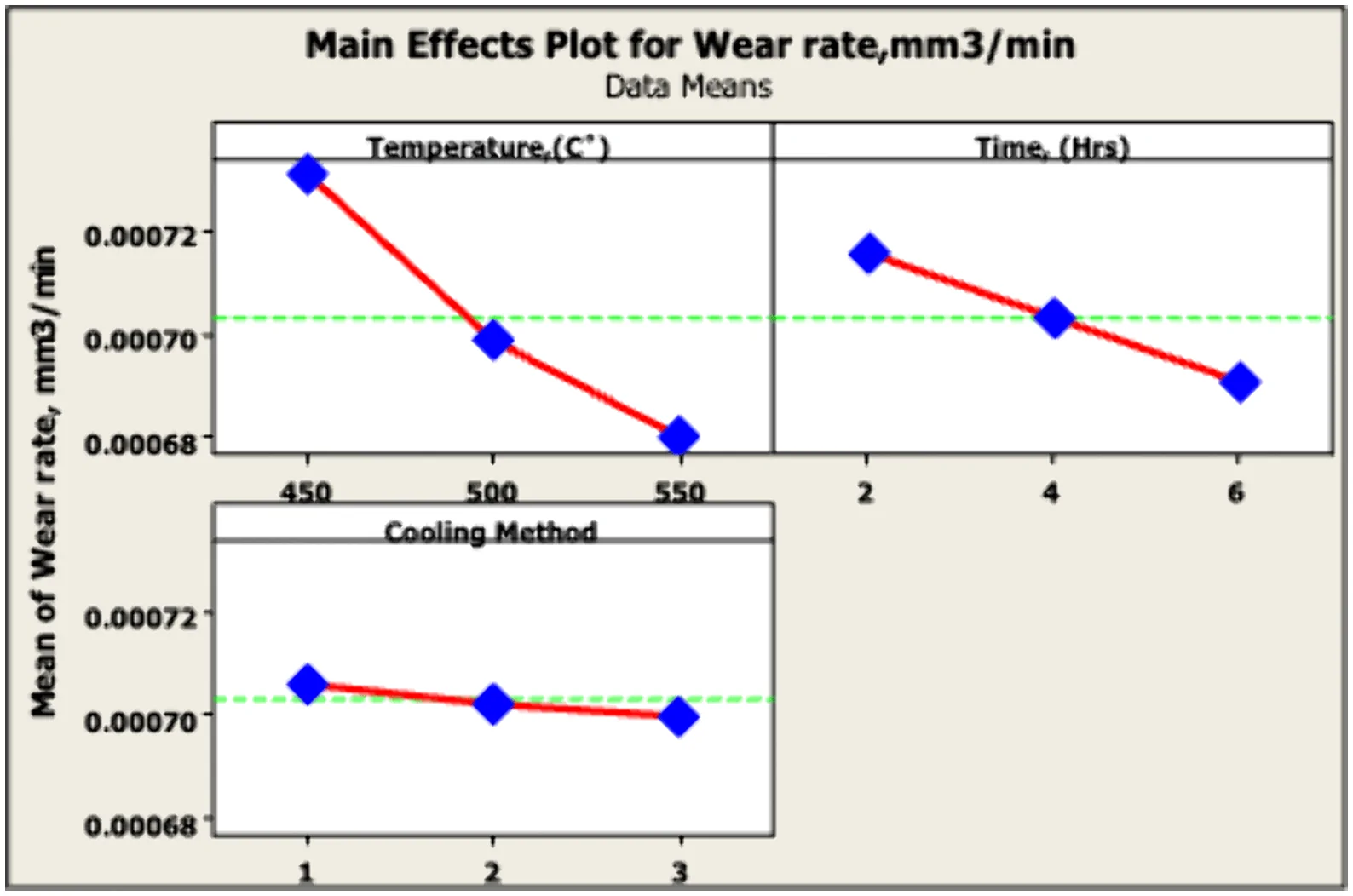
The main effect plots for the response of wear rate.

### Heat treatment effects on the responses of coefficient of friction (COF)

The coefficient of friction (COF) obtained during the wear tests ranged from 0.4002 to 0.4501, as summarized in Table 2. The minimum COF (0.4002) was recorded at 550 °C, 6 h and water quenching (Experiment 27), whereas the maximum value (0.4501) occurred at 450 °C, 2 h and furnace cooling (Experiment 1). The coefficient of friction decreased with increasing heat-treatment temperature and holding time within the investigated range. The presence of graphite may contribute to this reduction because graphite can act as a solid lubricant. However, post-wear surface characterisation was not performed, and the formation of a tribofilm or mechanically mixed layer cannot therefore be confirmed from the present results.

The main effects plot in Fig 6 indicates that solution treatment temperature exerted the greatest influence on the coefficient of friction, followed by holding time and cooling method. A gradual reduction in the average COF was observed with increasing temperature, suggesting that the selected heat treatment conditions improved the sliding behaviour of the developed composite. The corresponding experimental values are presented in Table 2.

**Fig 6:**
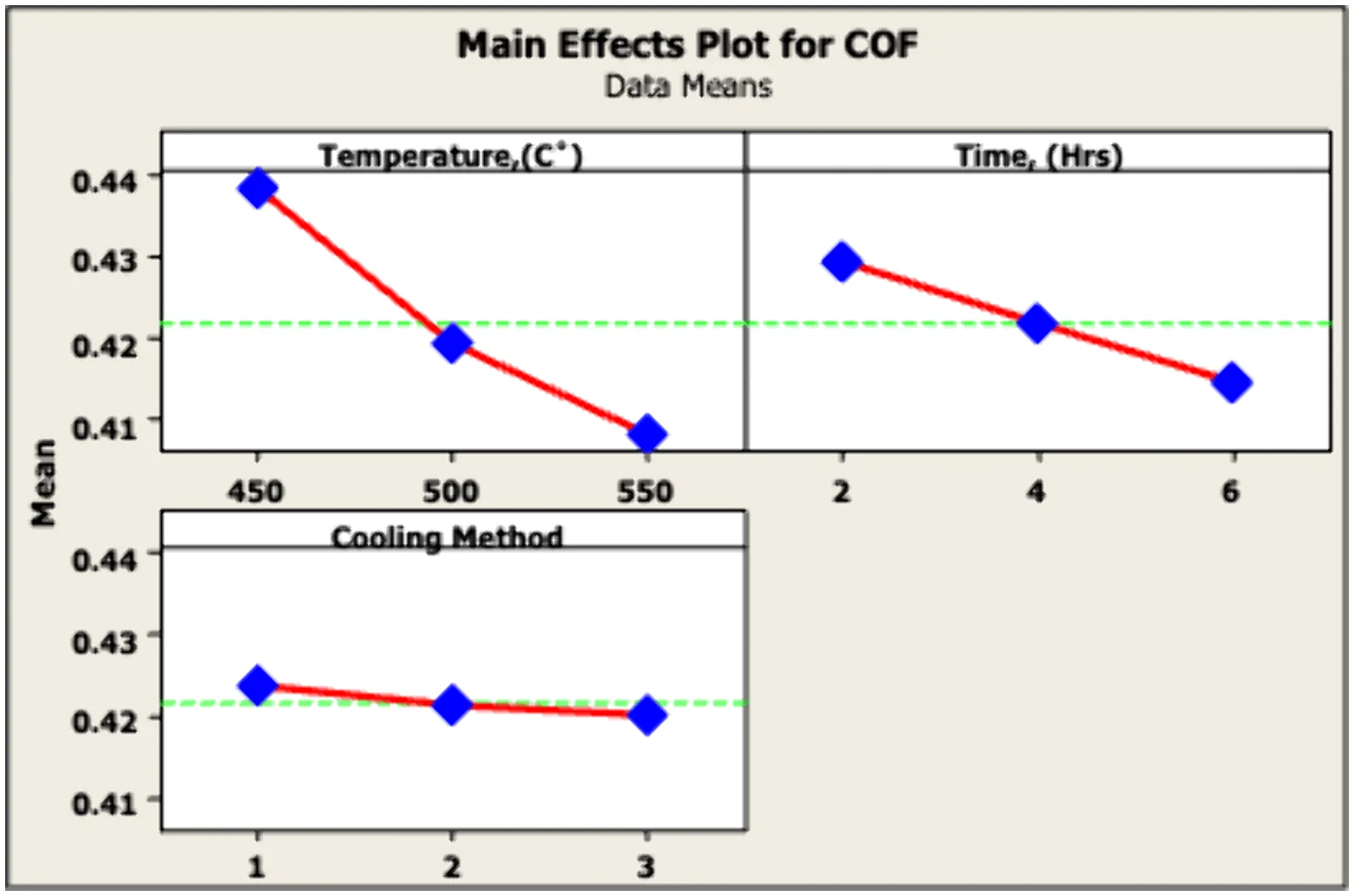
The main effect plots for COF.

The reduction in coefficient of friction is consistent with previous studies of graphite-containing aluminium composites, in which graphite has been associated with reduced friction through its solid-lubricating action [21,39]. However, transfer-film formation was not directly examined in the present study. The TOPSIS relative-closeness values for the 27 experimental conditions are presented in Table 3. Experiment 18, corresponding to 500 °C, 6 h, and water quenching (A2B3C3), obtained the highest relative-closeness value, 0.8933, among the tested trials. The TOPSIS main-effects plot in Fig 7 and the mean response values in Table 5 were then used to determine the factor-level combination. The highest mean TOPSIS relative-closeness values were obtained at 550 °C (A3), 6 h (B3), and water quenching (C3), giving A3B3C3 as the factor-level optimum within the investigated design space. This factor-level optimum differs from Experiment 18 because it was obtained by selecting the highest mean response at each factor level. Temperature showed the largest Max–Min value, followed by cooling method and holding time, indicating that temperature had the greatest main-effect range for the TOPSIS response.

**Fig 7:**
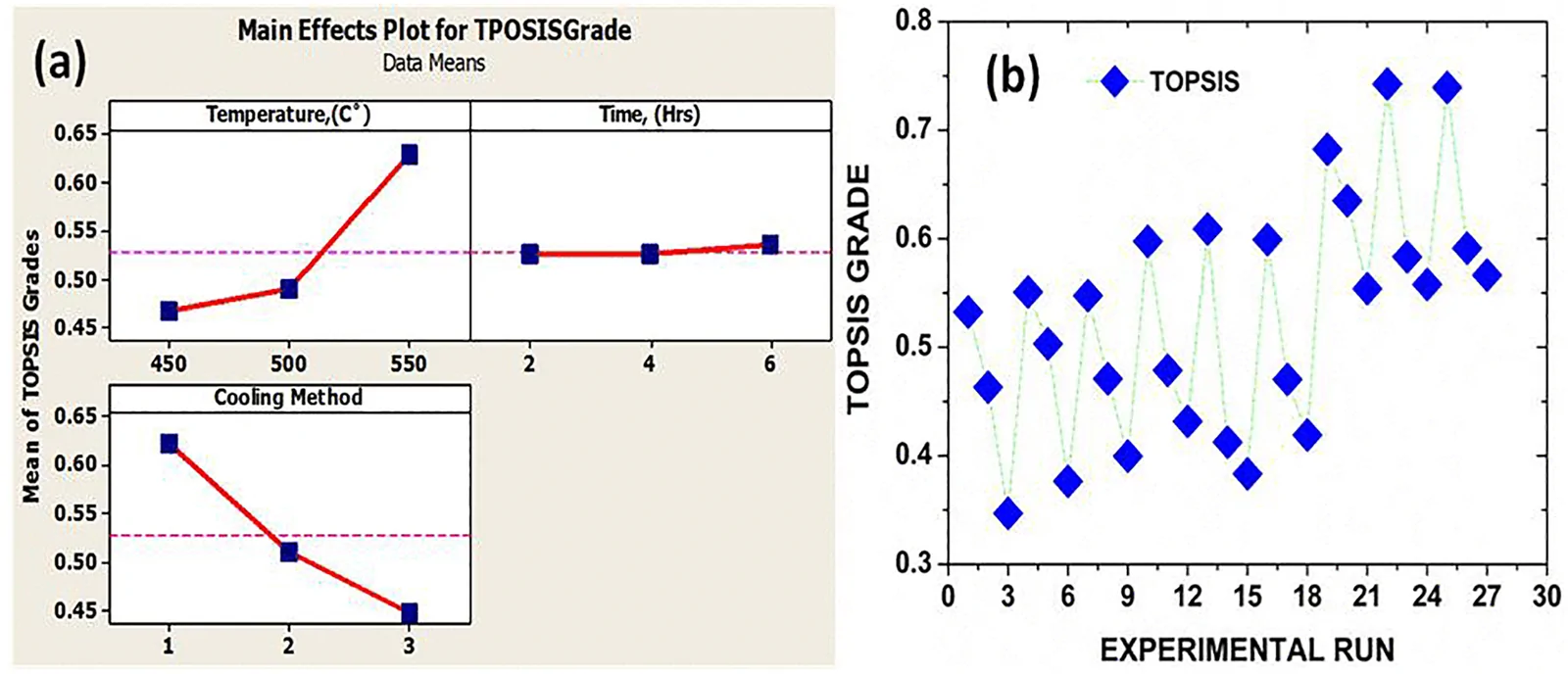
Main effects plot for TOPSIS relative closeness.

The mean TOPSIS relative closeness values at each factor level were calculated to determine the factor-level combination associated with the highest overall response. The results are presented in Table 4. Temperature showed the largest Max–Min value, followed by cooling method and holding time, indicating that temperature had the greatest effect on the TOPSIS response within the investigated parameter range.

**Table 4:** Response means of TOPSIS relative closeness for the heat treatment parameters.

Factor	Level 1	Level 2	Level 3	Max–Min	Rank
Temperature (°C)	0.3043	0.6196	0.639	0.3347	1
Holding time (h)	0.4036	0.5249	0.6344	0.2308	3
Cooling method	0.3831	0.546	0.6339	0.2508	2

The TOPSIS main effects plot (Fig 7) and the corresponding mean response values (Table 4) were used to determine the factor levels giving the highest average relative closeness. The highest mean TOPSIS values were obtained at 550 °C (A3), 6 h (B3) and water quenching (C3), giving A3B3C3 as the optimum factor-level combination within the investigated design space. Temperature showed the largest range among the three factors, followed by cooling method and holding time.

### Analysis of variance for the responses and for the TOPSIS Grade

The ANOVA model considered the three main heat treatment factors to evaluate their individual effects within the investigated parameter range. Interaction effects were not separately estimated, and the residual term includes variation not explained by the three fitted main effects and any interaction effects omitted from the model. Each of the 27 factor combinations is represented by one reported response value in the available experimental dataset. Consequently, independent within-condition variability and measurement repeatability cannot be estimated from the available data. The residual term in the main-effects ANOVA therefore represents variation not explained by the fitted factors and should not be interpreted as a direct estimate of experimental repeatability. The ANOVA results are summarised in Table 5. For hardness, cooling method was the most influential parameter, followed by temperature and holding time, with all three factors statistically significant (p < 0.001). For wear rate and coefficient of friction, temperature showed the greatest influence, followed by holding time and cooling method. These results are consistent with the factor-level analysis in Table 4, where temperature showed the largest range in the mean TOPSIS relative closeness, followed by cooling method and holding time.

**Table 5:** ANOVA for the measured responses of Hardness, Wear rate and COF & TOPSIS Grade.

Response	Factor	DoF	Sum of squares	Mean square	F-value	p-value
Hardness	Temperature	2	27.62407	13.81203	46.18	<0.001
	Holding time	2	16.05007	8.02503	26.83	<0.001
	Cooling method	2	57.92927	28.96463	96.84	<0.001
	Error	20	5.98187	0.29909	—	—
	Total	26	107.5853	—	—	—
Wear rate	Temperature	2	1.19756 × 10^−8^	5.98781 × 10^−9^	391.27	<0.001
	Holding time	2	2.81341 × 10^−9^	1.40670 × 10^−9^	91.92	<0.001
	Cooling method	2	1.66741 × 10^−10^	8.33704 × 10^−11^	5.45	0.013
	Error	20	3.06074 × 10^−10^	1.53037 × 10^−11^	—	—
	Total	26	1.52619 × 10^−8^	—	—	—
COF	Temperature	2	0.00429	0.002145	399.86	<0.001
	Holding time	2	0.001017	0.000509	94.8	<0.001
	Cooling method	2	6.44E-05	3.22E-05	6	0.009
	Error	20	0.000107	5.36E-06	—	—
	Total	26	0.005479	—	—	—
TOPSIS relative closeness	Temperature	2	0.63551	0.31775	129.99	<0.001
	Holding time	2	0.23985	0.11993	49.06	<0.001
	Cooling method	2	0.29156	0.14578	59.64	<0.001
	Error	20	0.04889	0.00244	—	—
	Total	26	1.21581	—	—	—

DoF, degrees of freedom; COF, coefficient of friction. The ANOVA model included the three main heat-treatment factors. Interaction effects were not separately estimated; therefore, the residual term includes variation not explained by the fitted main effects and any interaction effects omitted from the model. Accordingly, no separate experimental uncertainty or repeatability estimate is reported for the individual response measurements. A p-value below 0.05 was considered statistically significant. Values below 0.001 are reported as p < 0.001.

As presented in Table 5, the main-effects ANOVA indicates that all three heat-treatment parameters contributed to the variation in hardness, with cooling method exhibiting the highest F-value and therefore the largest modelled effect. For wear rate and coefficient of friction, solution-treatment temperature showed the largest modelled effect, followed by holding time and cooling method. For the TOPSIS relative closeness, solution-treatment temperature showed the greatest modelled effect, followed by cooling method and holding time. The ANOVA results are consistent with the trends observed in the main-effects plots (Figs 4–7). Because independent replicate measurements were not available, the residual term represents variation not explained by the fitted main-effects model and cannot be interpreted as a direct estimate of experimental measurement error or repeatability. Therefore, the F-values and associated p-values are interpreted as indicators of the relative effects within the fitted model rather than as independent estimates of experimental significance. The observed improvement in tribological performance is consistent with the combined influence of heat treatment and hybrid reinforcement. Solution treatment and subsequent cooling altered the condition of the aluminium matrix, resulting in changes in hardness and sliding behaviour. During sliding, graphite may contribute to friction reduction through its solid-lubricating action, while the harder fly-ash particles may assist in resisting material removal. These effects are consistent with the measured trends of increased hardness, reduced wear rate and lower coefficient of friction in the present investigation.

### Additional comparison of the selected heat-treatment condition

The Taguchi–TOPSIS analysis identified A3B3C3, corresponding to 550 °C, 6 h and water quenching, as the optimum factor-level combination within the investigated experimental range. This selection was obtained from the highest mean TOPSIS relative-closeness response at each factor level. Among the 27 experimental trials, Experiment 18 (A2B3C3), corresponding to 500 °C, 6 h and water quenching, produced the highest observed TOPSIS relative-closeness value of 0.8933. Because the available experimental dataset contains one reported response for each factor combination and does not include an independent replicate at the predicted optimum, a separate experimental confirmation of A3B3C3 could not be performed from the available data. Therefore, the present result is treated as a Taguchi–TOPSIS factor-level optimum within the investigated design space rather than as an independently validated optimum. The distinction between the factor-level optimum and the highest observed experimental run is important because the former is derived from the main effects, whereas the latter represents the response obtained for one specific experimental combination.

## Conclusions

This study investigated the influence of heat treatment parameters on the tribological performance of stir-cast LM26/graphite/fly ash hybrid aluminium matrix composites. A Taguchi L27 orthogonal array was employed to evaluate the effects of solution treatment temperature, holding time and cooling method. Multi-response optimization was performed using the TOPSIS technique, while ANOVA was used to identify the statistically significant heat treatment parameters. Based on the experimental results, the following conclusions are drawn.

- The Taguchi–TOPSIS analysis identified A3B3C3 (550 °C, 6 h and water quenching) as the optimum factor-level combination within the investigated experimental range for simultaneously considering hardness, wear rate and coefficient of friction.
- Experiment 18, corresponding to 500 °C, 6 h and water quenching, produced the highest individual TOPSIS relative-closeness value (0.8933) among the 27 experimental trials, whereas the factor-level analysis identified A3B3C3 as the optimum combination based on the mean response at each factor level. The two results should not be interpreted as independent confirmation of one another.
- The highest measured hardness was 51.2 BHN at 500 °C for 6 h with water quenching, while the lowest wear rate (0.000667 mm^3^/min) and coefficient of friction (0.4002) were obtained at 550 °C for 6 h with water quenching. The experimental values supporting these observations are reported in Table 2.
- The main-effects ANOVA indicated that cooling method had the largest modelled effect on hardness, whereas solution-treatment temperature had the largest modelled effect on wear rate and coefficient of friction. For the TOPSIS relative closeness, solution-treatment temperature showed the largest modelled effect, followed by cooling method and holding time. These results describe the relative effects within the fitted main-effects model; because independent replicate measurements were not available, the residual variation cannot be interpreted as a direct estimate of experimental repeatability.
- The combined heat treatment and hybrid reinforcement conditions investigated in this study resulted in measurable changes in hardness, wear rate and coefficient of friction. The results provide an experimental basis for selecting heat treatment conditions for the LM26/graphite/fly ash composite within the investigated parameter range.

The present investigation was limited to the selected solution heat-treatment parameters and dry-sliding tribological responses. The available experimental dataset contains one reported response value for each of the 27 factor combinations; therefore, independent experimental repeatability could not be quantified. Surface roughness measurements, artificial ageing, different reinforcement fractions, detailed phase evolution, residual stresses, post-wear surface and subsurface mechanisms, and long-term service performance were not investigated. Future work should include replicated testing, surface roughness measurement, XRD and EDS phase/chemical characterisation, post-wear SEM/EDS analysis, and extended tribological testing to clarify the mechanisms underlying the observed response trends.

## Supporting information

S1 Data Raw experimental data supporting the heat-treatment optimization of LM26/graphite/fly ash hybrid aluminium matrix composites. (PDF)
