# Supplementary material for: Effect of Heat treatment parameters on the tribological performance of LM26/graphite/fly ash hybrid aluminium matrix composites using Taguchi-TOPSIS optimization
Source: PLoS One. 2026 Oct 9;21(10):e0360156. doi: 10.1371/journal.pone.0360156 (PMC13652234; doi:10.1371/journal.pone.0360156)
Supplement: Supplementary file 1 [file pone.0360156.s001.pdf]

Supporting Information- S1 Dataset

Raw experimental dataset supporting the heat treatment optimization of LM26/graphite/fly ash hybrid aluminium matrix composites.

This supplementary dataset contains the complete experimental measurements obtained from the Taguchi L27 orthogonal array. The dataset was used for the TOPSIS optimization, analysis of variance (ANOVA) and confirmation experiments presented in the manuscript.

Table S1. Raw experimental data obtained from the Taguchi L27 orthogonal array for the heat treatment optimization of LM26/graphite/fly ash hybrid aluminium matrix composites. The table summarizes the experimental design factors and the corresponding measured tribological responses used in the statistical analyses presented in the manuscript.

Ex.No.	Temperature,(C°)	Time, (Hrs)	Cooling Method+	Hardness (BHN)	Wear rate,mm^3/min	COF
1	450	2	1	42.3	0.00075	0.4501
2	450	2	2	44.93	0.00074	0.444
3	450	2	3	46.96	0.000739	0.4434
4	450	4	1	43.8	0.000731	0.4387
5	450	4	2	45.1	0.000728	0.4366
6	450	4	3	47.23	0.000727	0.4362
7	450	6	1	44.49	0.000725	0.435
8	450	6	2	46.2	0.000721	0.4326
9	450	6	3	47.8	0.000715	0.429
10	500	2	1	44.72	0.000713	0.4278
11	500	2	2	46.8	0.000712	0.4272
12	500	2	3	47.8	0.000709	0.4254
13	500	4	1	45.3	0.000705	0.423
14	500	4	2	48.7	0.000703	0.4218
15	500	4	3	49.52	0.000701	0.4206
16	500	6	1	47.1	0.000683	0.4098
17	500	6	2	49.9	0.000679	0.4072
18	500	6	3	51.2	0.000681	0.4086
19	550	2	1	44.61	0.000699	0.4194
20	550	2	2	46.1	0.00069	0.414
21	550	2	3	47.5	0.000688	0.4128
22	550	4	1	45.2	0.000677	0.407
23	550	4	2	47.72	0.000677	0.4062
24	550	4	3	48.3	0.000675	0.405
25	550	6	1	45.5	0.000673	0.4038
26	550	6	2	47.91	0.000671	0.4026
27	550	6	3	48.6	0.000667	0.4002
† Cooling method: 1 = Furnace cooling; 2 = Air cooling; 3 = Water quenching.						

Variable descriptions

Experiment No.: Experimental run number.

Temperature (°C): Solution treatment temperature.

Holding Time (h): Solution treatment duration.

Cooling Method: 1=Furnace cooling; 2=Air cooling; 3=Water quenching.

Hardness: Measured hardness.

Wear Rate: Measured wear rate (mm3/min).

COF: Measured coefficient of friction.

All experiments were conducted according to the Taguchi L27 orthogonal array under identical testing conditions. Each row represents one experimental trial included in the statistical analysis.

Figure S1. Scanning electron microscopy (SEM) micrographs of the stir-cast LM26/graphite/fly ash hybrid aluminium matrix composite acquired at different magnifications (20×, 100×, 1K, 2K, 3K, 5K, 10K and 15K). The images confirm the distribution of graphite and fly ash reinforcement particles within the aluminium matrix and provide additional microstructural observations supporting the representative SEM image presented in Fig. 2 of the main manuscript.

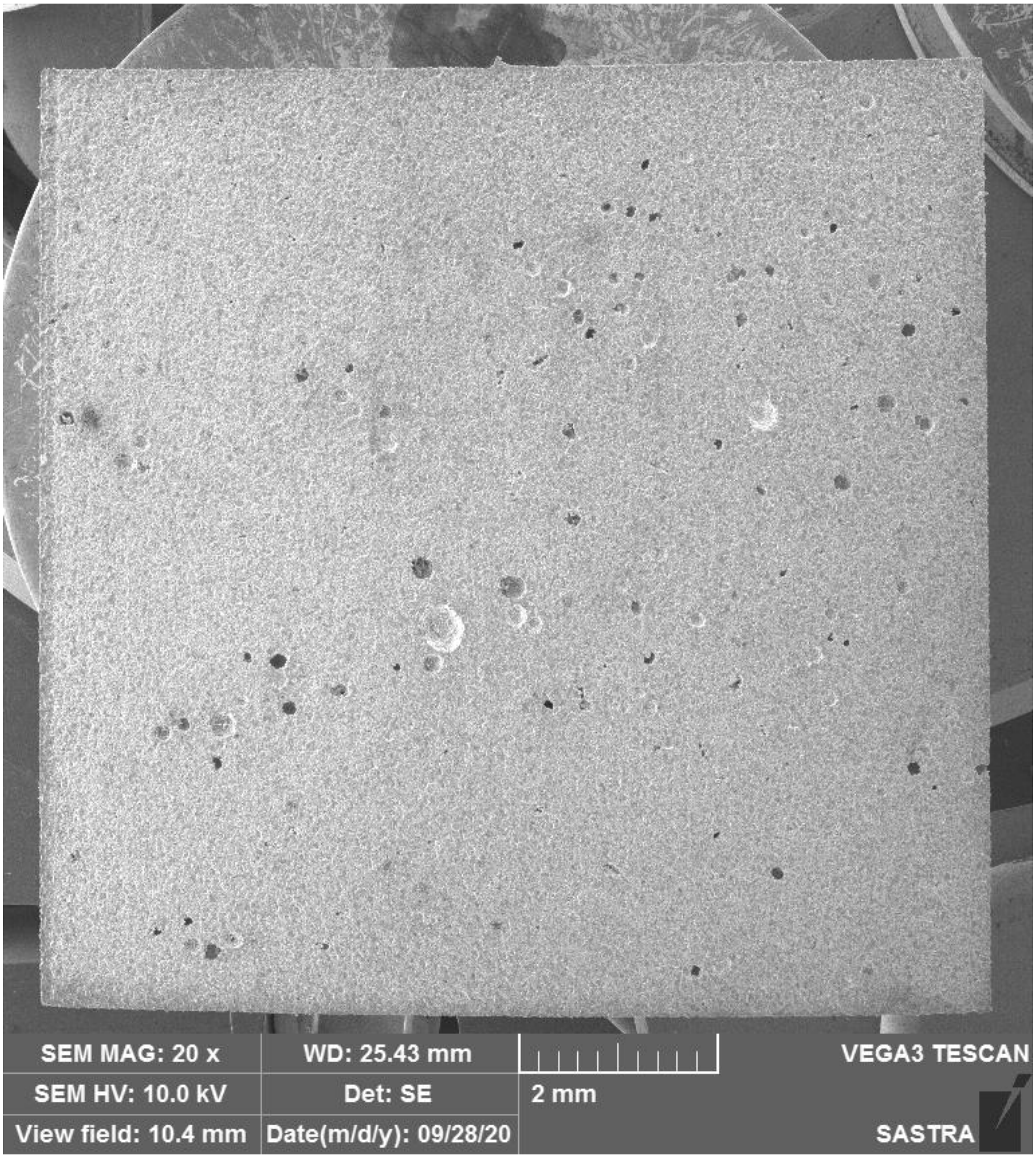
SEM MAG: 20 x
WD: 25.43 mm
VEGA3 TESCAN
SEM HV: 10.0 kV
Det: SE
2 mm
View field: 10.4 mm
Date(m/d/y): 09/28/20
SASTRA

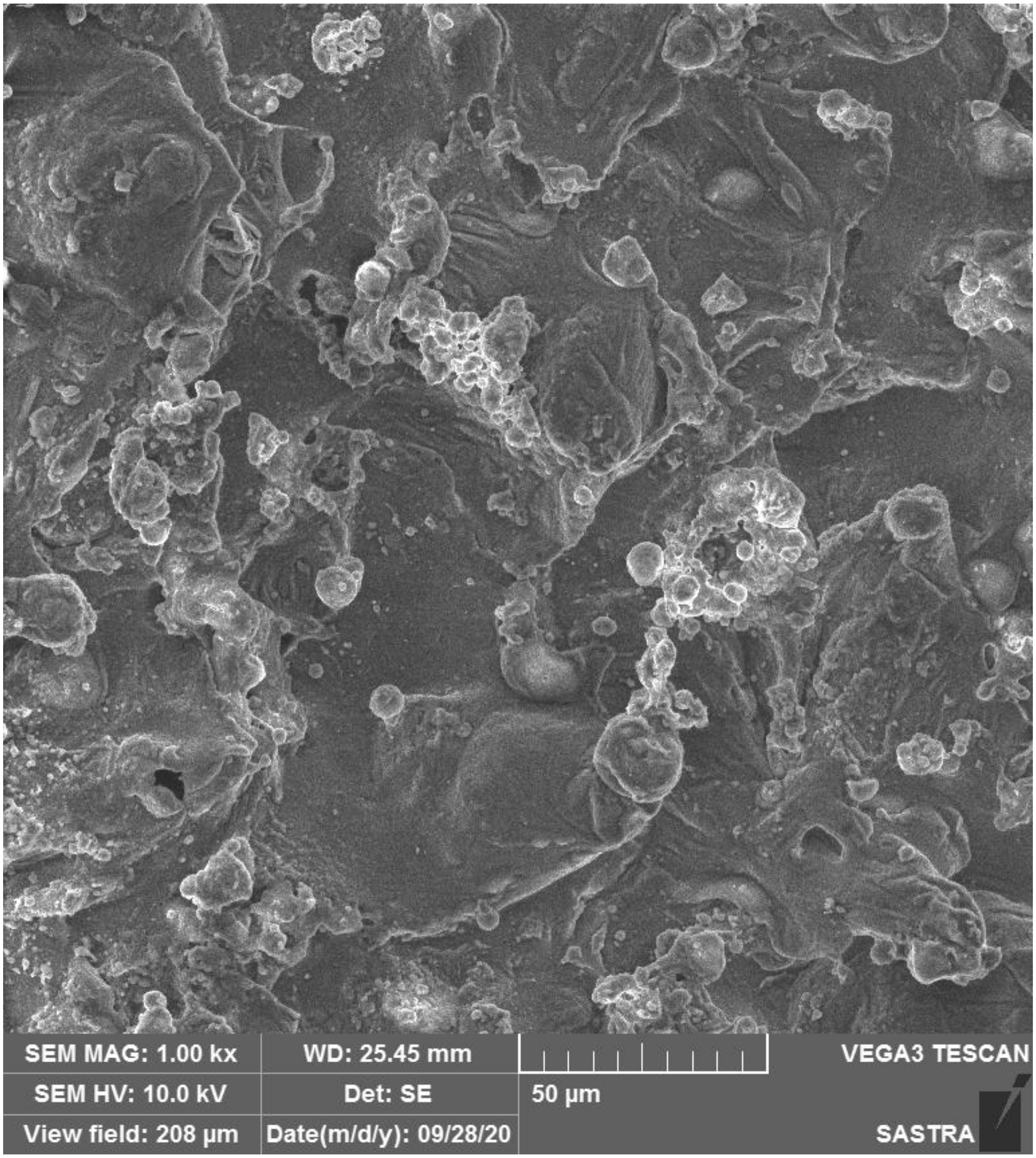
SEM MAG: 1.00 kx
WD: 25.45 mm
VEGA3 TESCAN
SEM HV: 10.0 kV
Det: SE
50 μm
View field: 208 μm
Date(m/d/y): 09/28/20
SASTRA

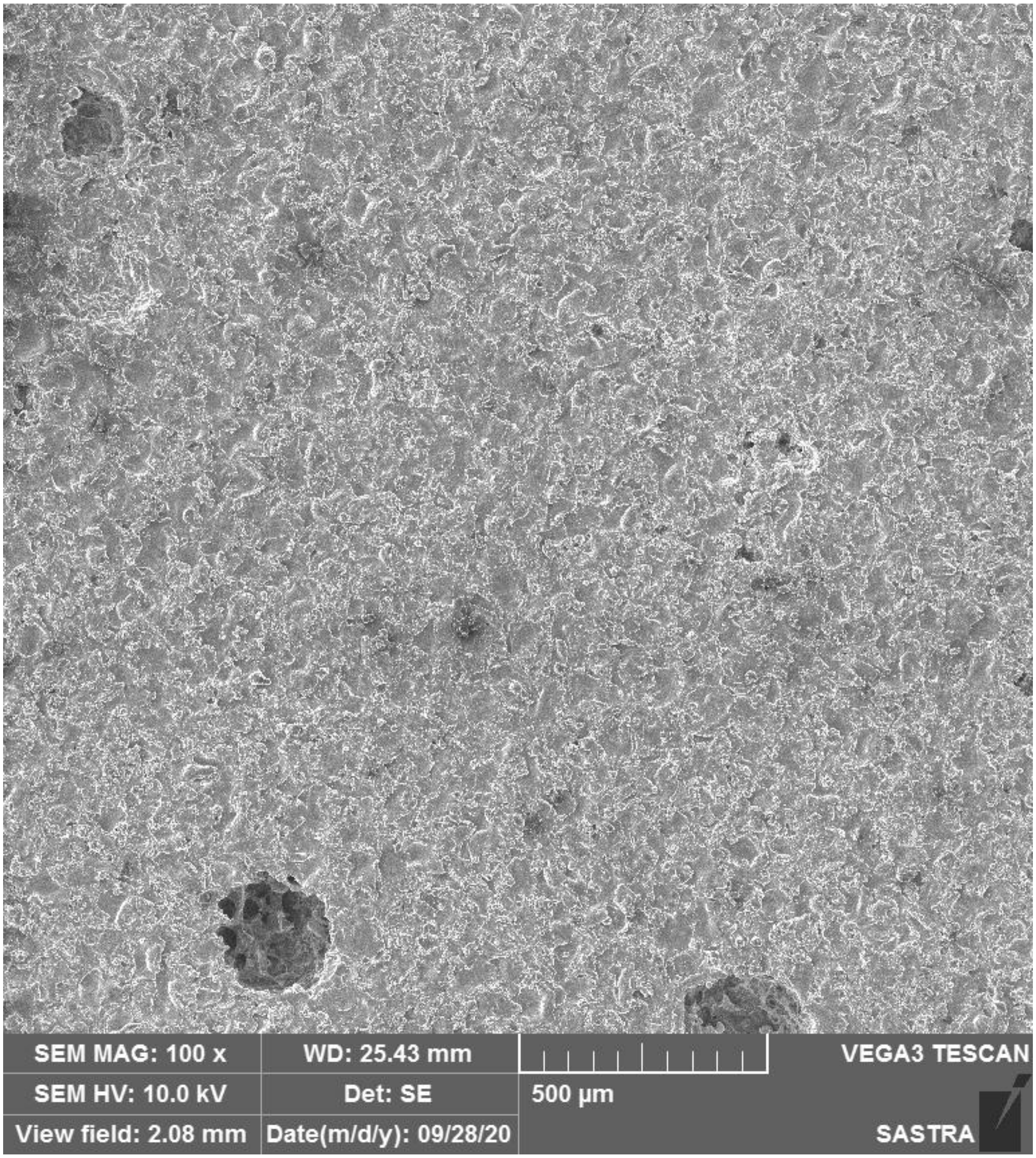
SEM MAG: 100 x
WD: 25.43 mm
VEGA3 TESCAN
SEM HV: 10.0 kV
Det: SE
500 µm
View field: 2.08 mm
Date(m/d/y): 09/28/20
SASTRA

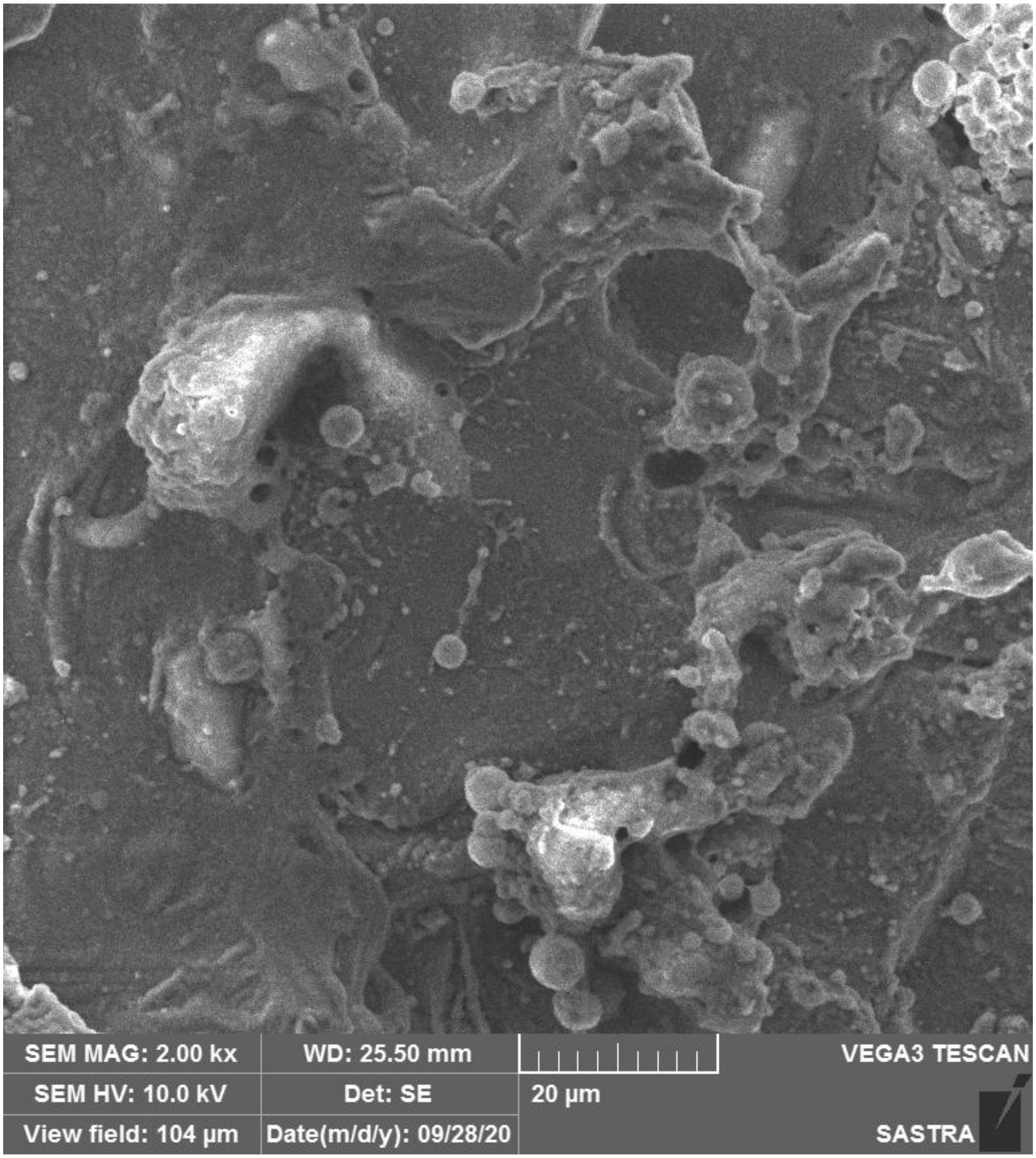
SEM MAG: 2.00 kx
WD: 25.50 mm
VEGA3 TESCAN
SEM HV: 10.0 kV
Det: SE
20 μm
View field: 104 μm
Date(m/d/y): 09/28/20
SASTRA

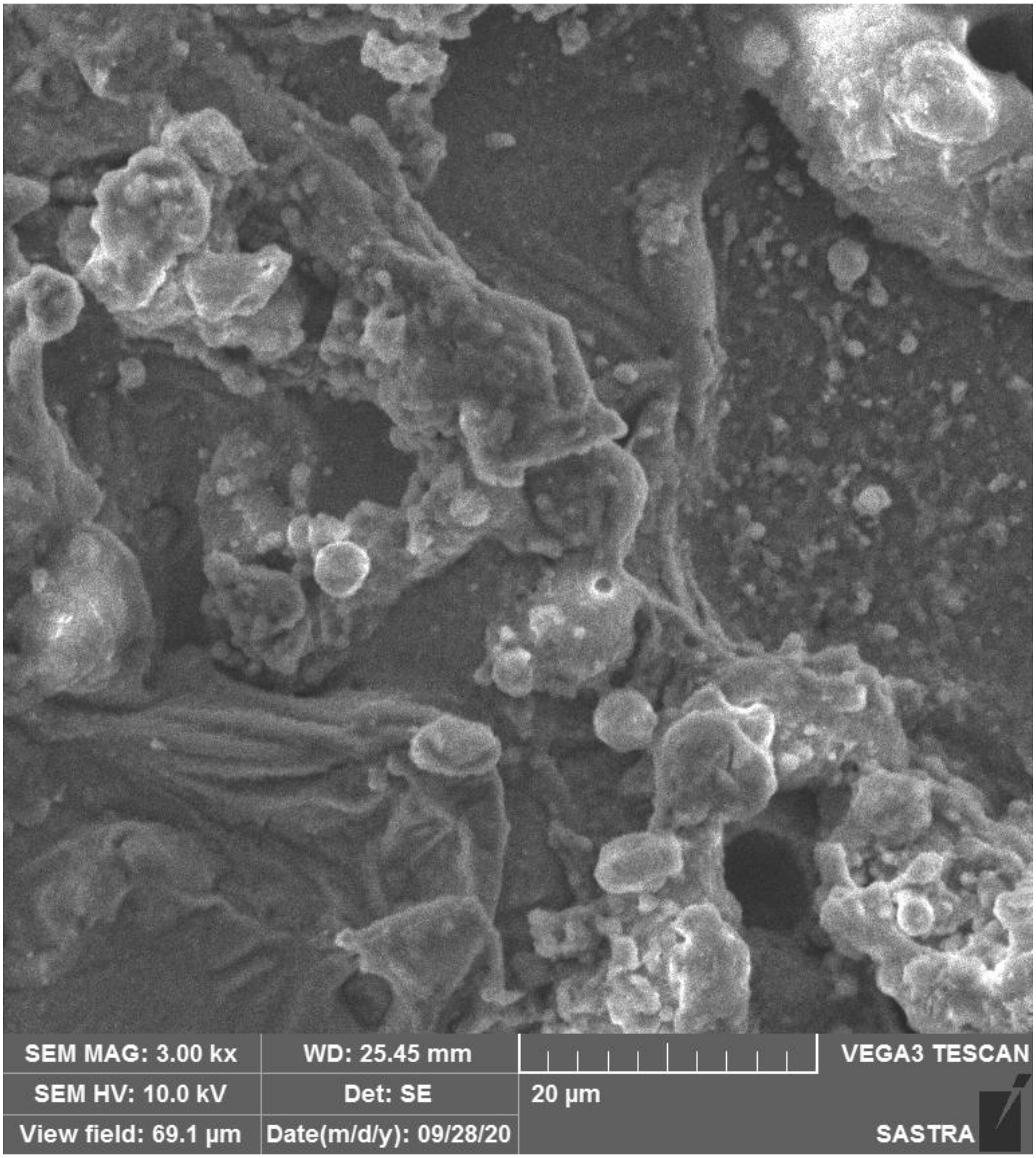
SEM MAG: 3.00 kx
WD: 25.45 mm
VEGA3 TESCAN
SEM HV: 10.0 kV
Det: SE
20 µm
View field: 69.1 µm
Date(m/d/y): 09/28/20
SASTRA

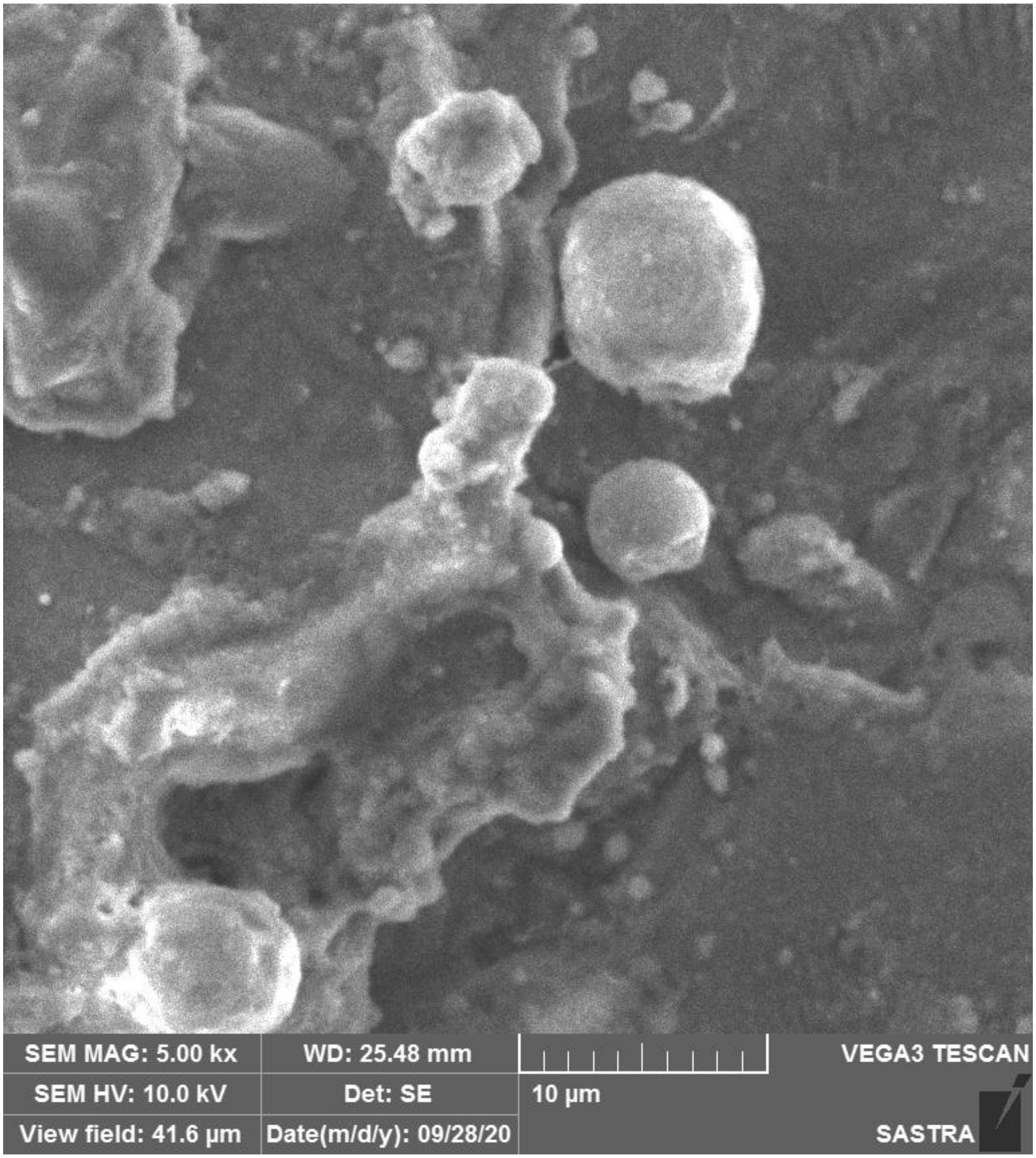
SEM MAG: 5.00 kx
WD: 25.48 mm
VEGA3 TESCAN
SEM HV: 10.0 kV
Det: SE
10 μm
View field: 41.6 μm
Date(m/d/y): 09/28/20
SASTRA

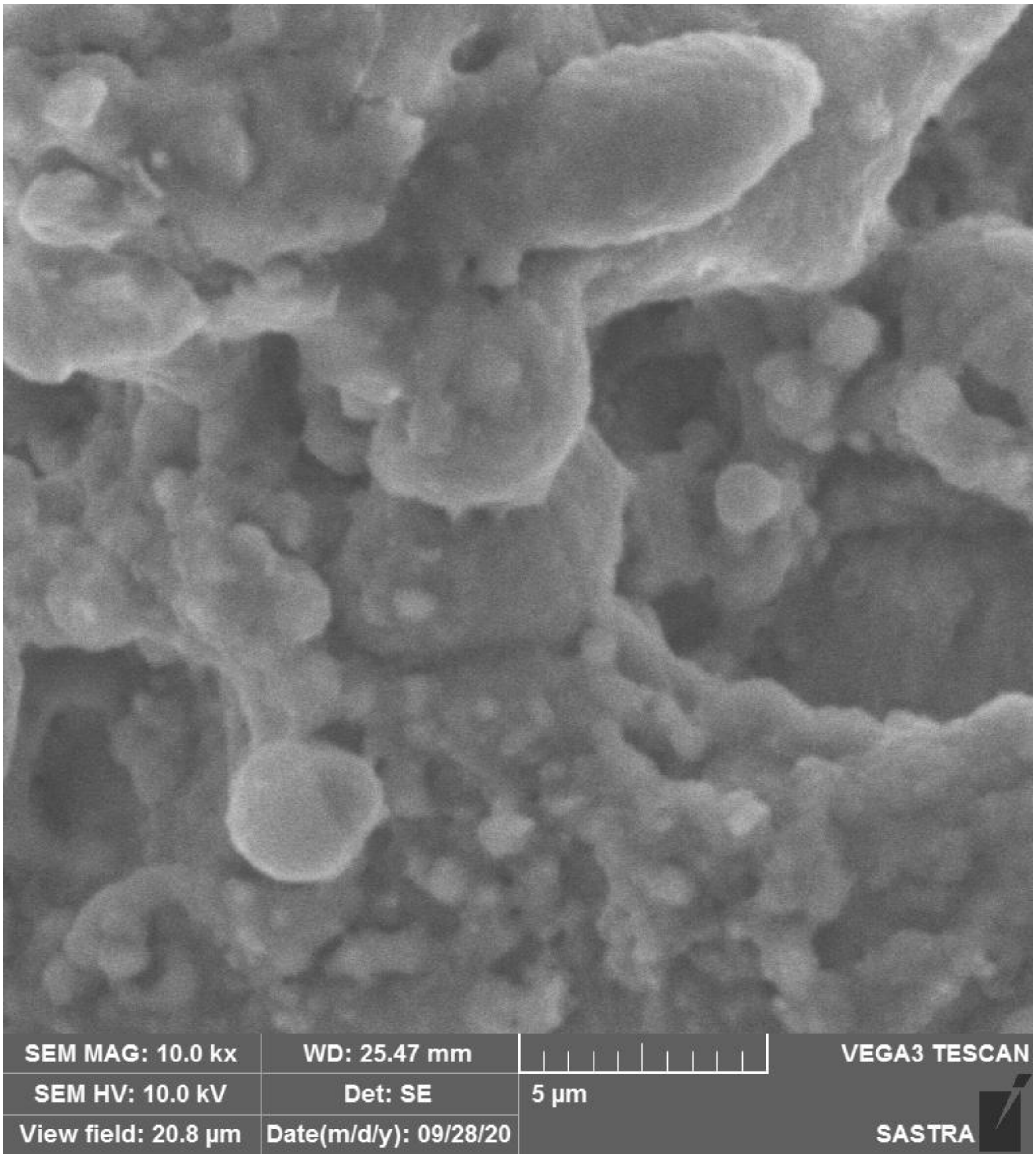
SEM MAG: 10.0 kx
WD: 25.47 mm
VEGA3 TESCAN
SEM HV: 10.0 kV
Det: SE
5 µm
View field: 20.8 µm
Date(m/d/y): 09/28/20
SASTRA

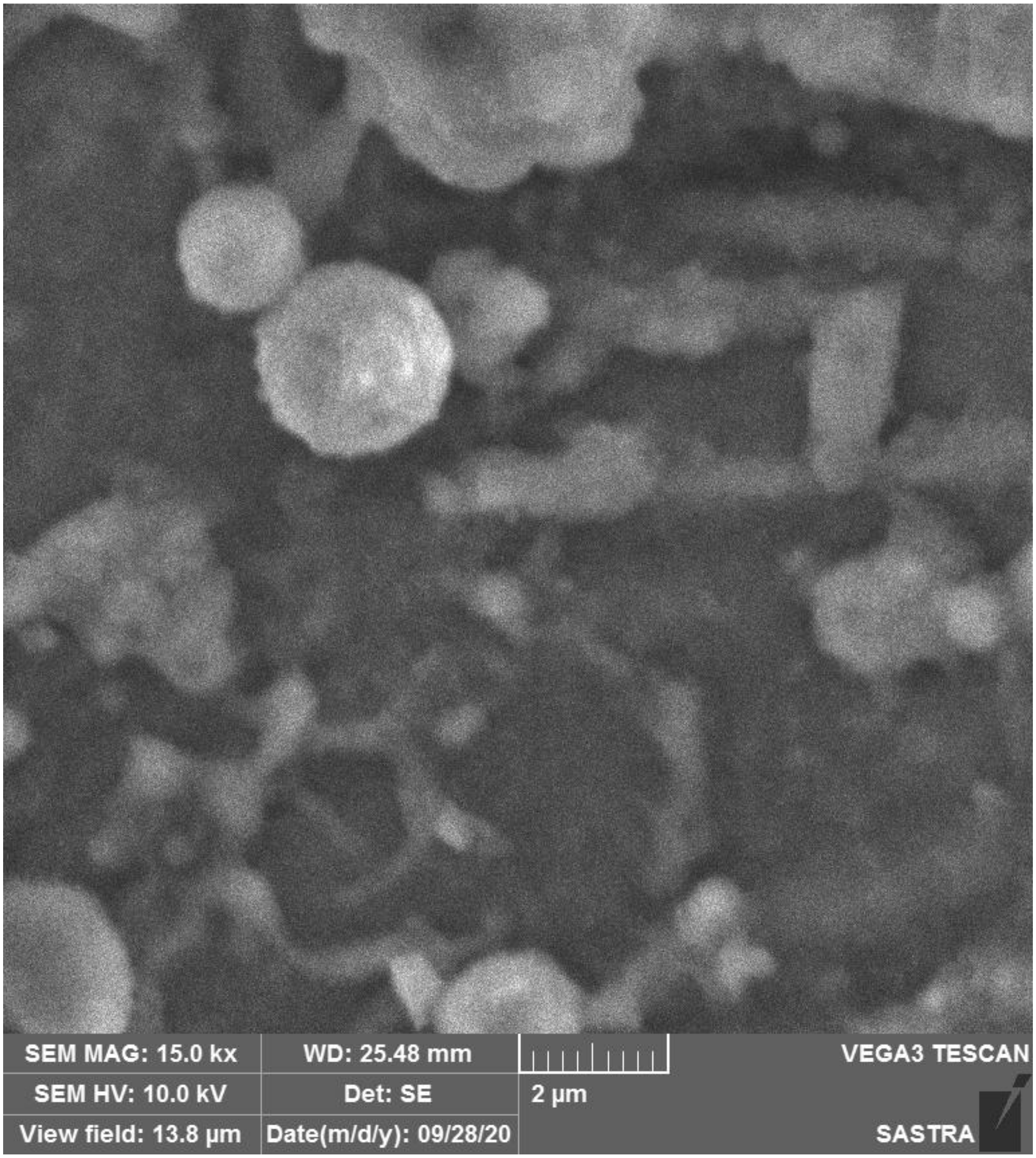

This supplementary dataset accompanies the associated manuscript and provides the complete experimental observations used in the statistical analyses reported in the main text.
